# On the shuttling across the blood-brain barrier via tubule formation: Mechanism and cargo avidity bias

**DOI:** 10.1126/sciadv.abc4397

**Published:** 2020-11-27

**Authors:** Xiaohe Tian, Diana M. Leite, Edoardo Scarpa, Sophie Nyberg, Gavin Fullstone, Joe Forth, Diana Matias, Azzurra Apriceno, Alessandro Poma, Aroa Duro-Castano, Manish Vuyyuru, Lena Harker-Kirschneck, Anđela Šarić, Zhongping Zhang, Pan Xiang, Bin Fang, Yupeng Tian, Lei Luo, Loris Rizzello, Giuseppe Battaglia

**Affiliations:** 1 School of Life Science, Anhui University, Hefei, P. R. China.; 2 Department of Chemistry, Anhui University, Hefei, P. R. China.; 3 Institute of Physical Science and Information Technology, Anhui University, Hefei, P. R. China.; 4 Department of Chemistry, University College London, London, UK.; 5 Institute for the Physics of Living Systems, University College London, London, UK.; 6 SomaNautix Ltd., London, UK.; 7 Sheffield Institute for Translational Neuroscience, University of Sheffield, Sheffield, UK.; 8 Institute of Cell Biology and Immunology, University of Stuttgart, Stuttgart, Germany.; 9 Department of Physics and Astronomy, University College London, London, UK.; 10 CAS Center for Excellence in Nanoscience, Institute of Intelligent Machines, Chinese Academy of Science, Hefei, China.; 11 College of Pharmaceutical Sciences, Southwest University, Chongqing, P. R. China.; 12 Institute for Bioengineering of Catalonia (IBEC), The Barcelona Institute for Science and Technology (BIST), Barcelona, Spain.; 13 Catalan Institution for Research and Advanced Studies (ICREA), Barcelona, Spain.

## Abstract

The blood-brain barrier is made of polarized brain endothelial cells (BECs) phenotypically conditioned by the central nervous system (CNS). Although transport across BECs is of paramount importance for nutrient uptake as well as ridding the brain of waste products, the intracellular sorting mechanisms that regulate successful receptor-mediated transcytosis in BECs remain to be elucidated. Here, we used a synthetic multivalent system with tunable avidity to the low-density lipoprotein receptor–related protein 1 (LRP1) to investigate the mechanisms of transport across BECs. We used a combination of conventional and super-resolution microscopy, both in vivo and in vitro, accompanied with biophysical modeling of transport kinetics and membrane-bound interactions to elucidate the role of membrane-sculpting protein syndapin-2 on fast transport via tubule formation. We show that high-avidity cargo biases the LRP1 toward internalization associated with fast degradation, while mid-avidity augments the formation of syndapin-2 tubular carriers promoting a fast shuttling across.

## Teaser

Tubule formation acts as cargo transport across the blood-brain barrier.

## INTRODUCTION

The human brain accounts for about 2 to 3% of the total body mass, and yet, it consumes up to 50% of the total intake of oxygen and glucose (*1*). Such a high energy demand is only possible because of a controlled gating of mass exchange with the body across a network of barriers that are phenotypically regulated by the brain cells. The most important of all gateways is the blood-brain barrier (BBB). This is the richest capillary network in the body that can effectively feed the brain components with about one capillary per neuron and about 10 to 15 μm of the average distance between one capillary to another (*2*, *3*). Capillaries are made of polarized endothelial cells connected via tight junctions. Brain endothelial cells (BECs) are conditioned by the neighboring brain cells to limit passive transport by forming impermeable tight junctions, lacking fenestrations, and expressing efflux transporters that protect the brain from harmful compounds (*3*–*5*). BBB dysfunctions are at the core of aging, neurological degeneration, stroke, and multiple sclerosis (*4*). The BBB makes the brain impermeable to most therapeutics, leading to a bottleneck in drug development (*5*).

BECs control the transport of small molecules, such as glucose and amino acids, by expressing specialized solute carrier transporters on both apical (blood) and basal (brain) membranes that pump molecules across one by one (*6*). BECs overexpress transferrin (*7*), insulin receptors (*8*), and low-density lipoprotein receptor–related protein 1 (LRP1) (*9*–*11*), and these receptors are often involved in shuttling their respective ligands into trafficking membrane–enveloped carriers across the cell via a process collectively known as transcytosis (*6*, *12*). Among these receptors, LRP1 is a critical motif highly expressed by neurons (*13*) and astrocytes (*14*), and it has been reported to bind to more than 40 ligands (*10*) undergoing rapid endocytosis with a half-life of less than 30 s (*10*, *15*). LRP1 has been associated with the blood-to-brain efflux of lactoferrin (*16*), receptor-associated protein (RAP) (*17*), and Kunitz protease inhibitor (KPI) domain–containing proteins (*18*). Transcytosis is an active transport involving the rearrangement of large membrane volumes, and although it has been investigated in detail in other barrier tissues (such as the epithelium), little is known about it in endothelial cells (*8*, *12*, *19*). Epithelial, and by analogy endothelial, transcytosis involves three steps: (i) endocytosis, a vesicular carrier emerges from one side of the membrane, typically involving clathrin or caveolin; (ii) trafficking, the carrier moves toward and fuses with the endolysosome network; and eventually (iii) exocytosis, a new vesicular carrier emerges from endolysosome, moves toward, and fuses with the opposite side of the plasma membrane (*8*). This sequence of events is viable in thick epithelial cells but often endothelium can be as thin as few hundreds of nanometers (*19*), and as such, the internal volume is too small to house the machinery associated with the three transcytosis steps. Furthermore, although there is growing evidence supporting the role of transcytosis at the BBB, particularly via LRP1, the mechanism that determines whether the receptor is to be sorted for transcytosis or for degradation in lysosomes remains still enigmatic.

One of the parameters that appear to influence the mechanism of transcytosis at BECs is avidity of the cargo (*20*–*23*). Using a Brain Shuttle platform targeting transferrin receptor at BECs, it has been shown that a monovalent construct is successfully sorted for transcytosis and colocalizes with narrow intracellular tubules, while a bivalent one is sorted for degradation exhibiting impaired transport along such tubules (*22*). Previous ultrastructural observations also reported the formation of “pores” or “channels” spanning endothelial cells referred to as transendothelial channels (TECs) (*24*). Bundgaard (*25*) reconstructed three-dimensional (3D) projections from serial sections of transmission electron micrographs of hagfish BECs, showing that intracellular membranes arising from transcytosis were rarely single vesicles but, instead, part of large multidimensional dendritic networks or “tubes.” Tubular networks and chains of vesiculo-vacuolar organelles (VVOs) were also reported in fenestrated endothelium (*26*). Despite being widely observed using electron microscopy, the molecular identity and the mechanism regulating the formation of these tubular structures are still not completely understood, especially on transcytosis mediated by LRP1. A piece of essential information missing from all these studies is the role of the membrane-sculpting proteins, most notably those comprising a Bin/amphiphysin/Rvs (BAR) domain (*27*). Syndapin-2 is a Fer-CIP4 homology–BAR (F-BAR) protein that senses and induces positive curvature on membranes (i.e., invaginations) and thus stabilizes tubular carriers (*28*, *29*) through the BAR domain. Apart from the BAR domain, syndapin-2 also contains an Src homology 3 (SH3) domain that binds to dynamin-2 and to the WASP/Scar family proteins that, ultimately, regulate actin filaments (*27*). Although syndapin-2 is ubiquitously expressed and associated with fundamental endocytic trafficking proteins, its functions in transcytosis at BECs are still to be unraveled.

Here, we elucidate the trafficking mechanism of LRP1 in BECs and correlate its transcytosis mechanism with syndapin-2 using both in vitro and in vivo models of the BBB. We use synthetic vesicles, polymersomes (POs), functionalized with LRP1 targeting moieties established to transverse the BBB to assess how multivalency, and hence binding avidity, controls LRP1-mediated transcytosis. We demonstrate that binding avidity controls transcytosis of LRP1 and further shed light on the mechanisms and dynamics of a unique mechanism of tubulation regulated by syndapin-2 on BECs.

## RESULTS AND DISCUSSION

### LRP1 trafficking across brain endothelium

**Fig. 1:**
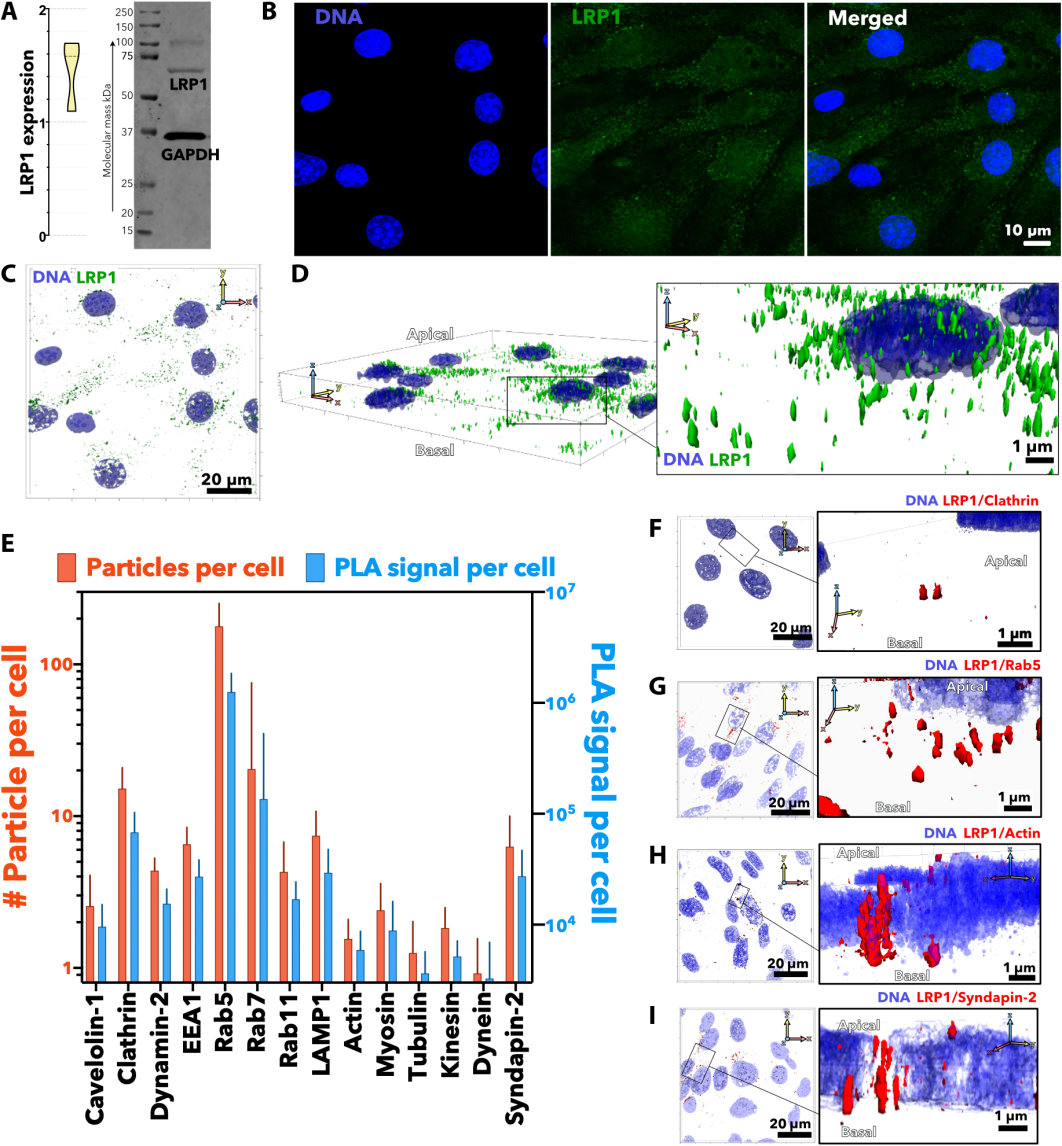
LRP1 intracellular mapping. Expression levels of LRP1 in BECs assessed by WB (**A**) and immunofluorescence (green) with cell nuclei counterstained with DAPI (blue) (**B**). 3D renderings of BECs with both DNA (DAPI in blue) and LRP1 [anti–immunoglobulin G (IgG) in green] labeled shown as top view (**C**) and projection (**D**). (**E**) PLA between LRP1 and several intracellular proteins associated with endocytosis and trafficking reported as number of PLA events per cell and the total PLA signal per cell. 3D rendering of BECs showing PLA events between LRP1 and clathrin (**F**), Rab5 (**G**), actin (**H**), and syndapin-2 (**I**).

To study the mechanism of LRP1-mediated transcytosis across BECs, we used a well-established 3D model of the BBB consisting of confluent mouse brain endothelioma cells (bEnd3) cultured onto collagen-coated porous transwell inserts (fig. S1A). We have established the barrier properties of this BBB model (*30*, *31*) by measuring both the transendothelial resistance (TEER) and an apparent permeability coefficient of different molecular mass dextran (*P*), calculated as

$$P=\frac{1}{C_{0}A}\frac{\mathrm{dQ}}{\mathrm{dt}}$$ (1)

where *C*_0_ is the initial cargo concentration, *A* is the total surface area of the transwell membrane, and $\frac{\mathrm{dQ}}{\mathrm{dt}}$ is the transport rate calculated as the gradient of mass over time. bEnd3 monolayers presented TEER values of ∼40 ohm**·**cm^2^, and for 4- and 70-kDa dextrans, we measured a permeability of *P*_4kDa_ = 19.6 and *P*_70kDa_ = 4.7 nm s^−1^, respectively (fig. S1B). bEnd3 monolayer presented a classical morphology with the expression of platelet endothelial cell adhesion molecule (PECAM-1) and tight junction proteins, claudin-5, and zona occludens 1 (ZO-1) (fig. S1C). Most relevant to the present study, we confirmed the expression of LRP1 in BECs using both Western blot (WB) (Fig. 1A) and immunofluorescence (Fig. 1B) targeting the cytosolic and extracellular domains, respectively. The micrographs collected across different monolayer regions show the wide expression of LRP1 in BECs (Fig. 1, B and C). Moreover, 3D reconstructions evidence that LRP1 is expressed on both the apical and basal cell surfaces as well as in the perinuclear area (Fig. 1D).

We next performed a proximity ligation assay (PLA) between LRP1 and various cellular components whose role has been reported during one or more stages of transcytosis (*8*, *12*). We evaluated the LRP1 association with early-stage endocytosis effectors (clathrin, caveolin-1, and dynamin-2), main cytoskeleton units (β-actin and α-tubulin), as well as their corresponding motors (myosin, kinesin, and dynein). We also assessed the interaction between LRP1 and early endosomes (Rab5 and EEA1), recycling endosomes (Rab11), late endosomes (Rab7), and lysosomes (LAMP-1). We then investigated the proximity between LRP1 and syndapin-2, the F-BAR domain–containing protein that can stabilize tubular structures. We performed the assay on polarized BECs by imaging them using confocal laser scanning microscopy and collecting about 20 optical sections. The images were then analyzed using an ad hoc developed algorithm to extract two parameters, the total PLA signal per cell (PLA_C_) and the number of events (*N*_E_) per single cell (Fig. 1E). The PLA_C_ quantifies whether we have interaction between LRP1 and the targeted protein and estimates the level of such an interaction. The *N*_E_, on the other hand, gives us an idea of whether the interaction is distributed across the cell or concentrated in particular loci. Ultimately, we used the confocal optical sections to generate 3D rendering (Fig. 1, F to I) of the proximity spots to reveal their morphology. The results in Fig. 1E show not only an evident and expected correlation between LRP1 and most of the endocytic cellular components but also a particularly strong association with clathrin, Rab5, and syndapin-2. The 3D rendering showing the proximity spots between LRP1 and clathrin in Fig. 1F reveals small spots with size at the limit of the confocal resolution, but with morphology suggesting small trafficking vesicles budding out. Similarly, the 3D rendering between LRP1 and Rab5 (Fig. 1G) resembles endosome morphology and size. We also observed a good association between LRP1 and β-actin, but little or no interaction with α-tubulin and all the molecular motors. The LRP1/actin association is concentrated in few events per cell, and the proximity spots appear as large tubules spanning almost the entire cell thickness (Fig. 1H). Similar structures across the cell were also observed between LRP1 and syndapin-2 (Fig. 1I), confirming their association into tubular membrane structures (*32*). On the basis of these results of proximity between LRP1 and syndapin-2 in in vitro BECs, we then demonstrated that syndapin-2 is expressed in vivo and is found in several brain cells including BECs (fig. S2, A to C). To interrogate tube dimensions and morphology, we then imaged brain sections in super-resolution using stimulated emission depletion (STED) microscopy to have a spatial resolution close to 50 nm. The detailed reconstruction of a single lectin-stained brain capillary is shown in fig. S2D, and it is evident that syndapin-2 is associated with tubular structures of diameter between 200 and 500 nm and lengths up to a few micrometers spanning across the endothelial cell (fig. S2, E and F). Hence, we established the association of LRP1 not only with vesicular endocytic proteins but also with syndapin-2 at BECs.

### Effect of avidity on BBB crossing

To elucidate how LRP1 moves across the BBB and regulates cargo sorting as a function of avidity, we used one of its most established ligand: angiopep-2. This peptide was derived from the LRP1-binding aprotinin (*18*) and demonstrated to cross the BBB shuttling anticancer drugs, (*33*), analgesics (*34*), RNA (*35*), DNA (*36*), and bacteriophage (*37*). We demonstrated in both mice (*30*) and rats (*38*, *39*) that when angiopep-2 is conjugated multivalently to the surface of pH-sensitive POs, it augments BBB crossing and enables the intracellular delivery of whole antibodies (*30*) and neuroprotective peptides (*39*) into central nervous system (CNS) cells. POs are synthetic vesicles formed by the self-assembly of block copolymers in water (*40*) whose final shape, size, and surface topology can be controlled bottom-up (*41*, *42*). As shown in fig. S3, we produced POs by mixing pristine (fig. S3A) and angiopep-2–modified poly[oligo(ethylene glycol) methacrylate]–poly [2(diisopropylamino)ethyl methacrylate] (POEGMA-PDPA) (fig. S3B) to make formulations displaying on their surface a different number of ligands and hence with different overall avidities (fig. S3C). The POs referred to here as A*_L_*-P, with *L* being the average angiopep-2 ligand number per particle, were produced to be the same size and morphology, as confirmed by both dynamic light scattering (DLS; fig. S3, D and E) and transmission electron microscopy (TEM) (fig. S3F). We can also infer that within the range of ligand number we used here, POs have almost identical surface chemistry as the highest ligand number *L* = 220, which corresponds to a 12% molar fraction of peptide-modified copolymers occupying 6% of the PO external surface area. Effectively, POs become the ideal cargo model to study the shuttling mechanisms across the BBB. To track the POs in vitro and in vivo, we conjugated cyanine 5 (Cy5) and Cy7 dyes to POEGMA-PDPA copolymers and mixed them to create the different formulations with a constant concentration of dye. Furthermore, we encapsulated an ad hoc synthesized 7-(*p*-tolyl)-5,6,8,9-tetrahydrodibenzo[c,h]acridine complexed with platinum and dimethyl sulfoxide (PtA2), of which synthesis mechanism and characterization are shown in fig. S4. This compound was chosen for its superior photostability and metallic nature, which allows imaging in super-resolution (STED) and TEM and also permits precise quantification in tissues by inductively coupled plasma mass spectrometry (ICP-MS) (*43*, *44*).

**Fig. 2:**
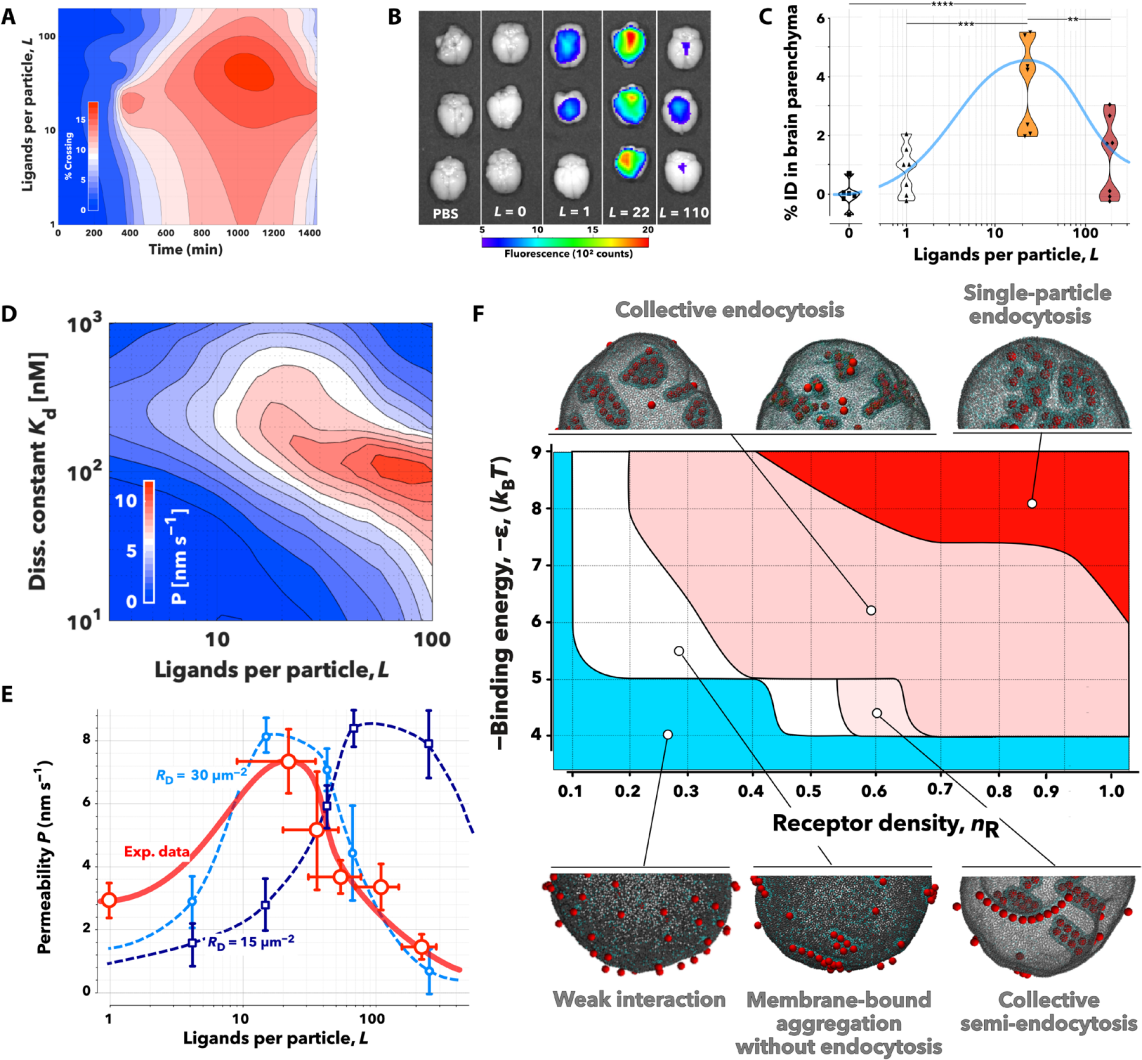
Ligand avidity versus BBB crossing. (**A**) Heatmap showing the experimental measurement of % of A*_L_*-P crossing as a function of incubation time and ligand number per particle (*L*). (**B**) Ex vivo fluorescent photographs of whole murine brains imaged 2 hours after intravenous injection of PBS, pristine POs (*L* = 0), free angiopep-2 peptide (*L* = 1), A_22_-P, or A_110_-P. Violin plots showing the quantification in the brain parenchyma of the various preparations tested. **P < 0.01, ***P < 0.001, and ****P < 0.0001, one-way ANOVA (*n* = 6). (**C**) Concentration of angiopep-2 functionalized cargo expressed as percentage of injected dose (% ID) per gram of tissue as a function of the number of ligands. (**D**) Heatmap of the apparent permeability, *P*, obtained from agent-based simulations as a function of the ligand number per particle and the single ligand dissociation constant, *K*_d_, with the LRP1 receptor. (**E**) Comparison between apparent permeability, *P*, across BBB experimental data (red markers and solid line) and simulation (blue markers and dashed lines) calculated for two different receptor densities and single ligand dissociation constant, *K*_d_ = 300 nM. Note that the control pristine PO apparent permeability was subtracted to the other formulations to remove passive diffusion. (**F**) Phase diagram showing different regimes of nanoparticle aggregation across the receptor densities and nanoparticle-receptor affinities expressed in *k*_B_*T* (with *k*_B_ being the Boltzmann constant and *T* the temperature) as observed in MD simulations. Nanoparticle distributions are illustrated MD simulations using a coarse-grained membrane surface patch.

We started by measuring the crossing of A*_L_*-P with *L* = 0, 22, 36, 56, 110, and 220 as well as the free angiopep-2, referred to here as *L* = 1, by administration to the apical compartmental of the in vitro BBB model and by quantifying the concentration of POs in the basal compartment. Data are collectively shown as a heatmap of crossing efficiency (%) as a function of ligand numbers per particle and incubation time (Fig. 2A). It is evident that BBB crossing does not linearly correlate with ligand number, and optimal crossing is shown at *L* = 22, with lower or higher ligand numbers showing a significantly reduced efficiency. From the in vitro screening, we selected three A*_L_-*P formulations: *L* = 0, 22, and 110 and the free peptide *L* = 1 for further testing in mice. After 2 hours of intravenous injection, we perfused the animals with saline solution to remove the excess of blood, harvested the whole brains, and imaged them using the in vivo imaging system (IVIS) (Fig. 2B). The same brains were subsequently processed to extract the parenchymal fraction and then to quantify the percentage of injected dose (% ID) of A*_L_-*P present in the tissue using near-infrared fluorescence spectroscopy (Fig. 2C). Both methods show that while all angiopep-2 formulations enter the brain and can be found at relatively high concentrations across the BBB, the formulation with the most effective crossing is again A_22_-P, in agreement with the in vitro data. Such a nonlinear dependence of the ligand binding energy on BBB crossing rate correlates with the results described for the targeting of transferrin receptor (*20*–*22*) and glucose transporter-1 (GLUT1) (*23*).

From a theoretical standpoint, transcytosis involves five major stages: binding, endocytosis, trafficking, exocytosis, and unbinding. Efficient transcytosis requires the formation of ligand/receptor bonds that last enough for it to be trafficked across; yet, the higher the ligand binding energy, the lower is its ability to detach once across to the other side. Therefore, a balance is required to form and maintain not only sufficiently strong bonds to enable binding and endocytosis but also a sufficiently weak bond to allow unbinding and release. Such an approximation allows the creation of an in silico model to stimulate transcytosis (see the Supplementary Materials). We used flexible large-scale agent-based modeling environment (FLAME), a generalized agent-based modeling platform, that models the behavior of individual POs undergoing Brownian motion, binding to endothelial cells, crossing the cells by transcytosis, and being released into the basal compartment (*45*). We designed the model based on the geometry of the transwell insert used in the in vitro experiments (fig. S1A), and the BECs were modeled as a uniform 2-μm-thick layer at the top of the insert. We also modeled POs with different ligand numbers and different individual ligand-receptor dissociation constants, starting them at time zero in the aqueous apical phase. POs were subjected to Brownian motion and bound to cells according to the multivalent-avidity binding model described in the Supplementary Materials. The particles were allowed to go through the different stages of transcytosis as described in fig. S5A, and the number of POs that crossed the BECs was measured. We thus used Eq. 1 to calculate the apparent permeability and plotted it as a function of both ligand number per particle (*L*) and the single ligand/receptor dissociation constant (*K*_d_). We used models for nanoparticles with radius *R*= 20 and 50 nm, as well as receptor densities *R*_D_= 15 and 30 μm^−2^, respectively. According to the simulations, there is a nonlinear dependence between ligand number and binding strength (fig. S5B), whereby the optimal transcytosis is obtained in a “Goldilocks” regime of avidity, i.e., not too strong and not too weak, and it is independent of the particle size or receptor density. We selected both size and receptor density to match our in vitro experimental data, and within such a range, our simulations suggest that bigger particles and larger receptor density lead to improved transcytosis. In Fig. 2D, we plot the apparent permeability across the BBB as a function of ligand numbers per particle (*L*) and the dissociation constant of the ligand/receptor binding (*K*_d_) for particles with radius *R* = 50 nm and receptor density *R*_D_ = 30 μm^−2^, which is very close to what we recently estimated using the super-selective theory (*46*). We know from previous work that angiopep-2 has a dissociation constant *K*_d_ = 313 nM (*47*), and using this, we can thus compare the simulations at similar dissociation constant with the experimental data. In Fig. 2E, we plot the experimental apparent permeability (in red), measured from the data in Fig. 2A and the simulations with *K*_d_ = 300 nM, particle with size *R* = 50 nm, and receptor densities *R*_D_ = 30 μm^−2^ or *R*_D_ = 15 μm^−2^. The experimental and simulation data show broad agreement, and the Goldilocks avidity effect is reproduced experimentally at similar values to those we observed computationally.

Last, we complemented both computational and experimental permeability measurements by performing molecular dynamics (MD) simulations to capture the effect of avidity on membrane topological changes and nanoparticle aggregation dynamics. We used a well-established coarse-grained membrane surface patch (fig. S5C) on an equilibrated spherical membrane and varied the receptor density and the nanoparticle to membrane binding energy (ϵ) expressed in *k*_B_*T*, with *k*_B_ being the Boltzmann constant and *T* the temperature. The latter represents the depth of the potential well in the attractive interaction between nanoparticles and “receptor” membrane beads (see Eq. 3 in the Supplementary Materials). A different initial nanoparticle distribution was randomly chosen for each simulation, and each parameter pair used the same set of six different initial nanoparticle distributions. The receptor density was represented in the model by the ratio of receptor membrane beads to the total number of membrane beads. The simulation results are summarized in Fig. 2F, and it is evident that across all receptor densities, no clear binding of the nanoparticles was observed for low binding energy. Some receptor beads clustered around individual adsorbed nanoparticles, but the nanoparticle-receptor adhesion was too weak to drive any interaction. As the binding energy increases, the nanoparticles bind to the membrane. While their relative adhesion energy is converted into membrane deformation, this is not sufficiently strong to induce full endocytosis. Nonetheless, progressively more particles bind the associated membrane deformations forming linear aggregates. The anisotropic aggregation is the consequence of the trade-off between nanoparticle-receptor adhesion and the membrane’s resistance to deformation (*48*). Higher binding energy results in the linear aggregates that can be internalized as tubular aggregates. These can coexist on a membrane together with membrane-bound tubular aggregates and internalized tubular aggregates. At higher receptor densities, lower binding energies are required for the nanoparticles to form tubular and linear aggregates. However, at high receptor density and high binding energy, the particles have sufficient adhesion to create singular deformation and enter via discrete endocytic events. The pseudo-phase diagram in Fig. 2F shows the limits of the different regimes observed in the simulations and the collective processes leading to different outcomes. As shown in fig. S5D, tubulation results in a high number of cargo units transported per single event, while higher binding energy and receptor density correspond to fewer number of particles per internalized carrier. The latter process is more efficient in internalizing the nanoparticles reaching almost 100%, while the collective process that occurs at lower binding energy and receptor density achieves only a much lower percentage of internalization (fig. S5E). Together, the MD simulations add another dimension to the avidity effect, showing that different binding energies drive alternative membrane deformations including tubulation and that these lead to a different endocytic initiation.

### Involvement of syndapin-2 in transcytosis as a function of avidity

**Fig. 3:**
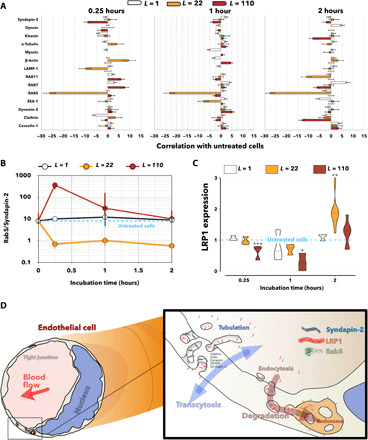
LRP1 subcellular localization and expression as a function of avidity. (**A**) Deviation of the number of proximity events measured by a PLA between untreated endothelial cells and treated for 0.25, 1, and 2 hours of incubation with free angiopep-2 peptide, *L* = 1, and A*_L_*-P, with *L* = 22 and *L* = 110. Note that zero corresponds to no variation, while positive and negative values indicate up- and down-regulation, respectively. (**B**) Ratio between LRP1/Rab5 and LRP1/syndapin-2 number of proximity events for the different treatments with free angiopep-2 peptide, *L* = 1, and A*_L_*-P, where *L* = 22 and *L* = 110, with Rab5/syndapin-2 being 10 for the untreated cells. (**C**) WB measuring the LRP1 expression relative to the untreated cells for free angiopep-2 peptide, *L* = 1, and A*_L_*-P, with *L* = 22 and *L* = 110 measured at different incubation times with 0.25, 1, and 2 hours. *P < 0.05, **P < 0.01, and ***P < 0.001, one-way ANOVA (*n* = 6). Note that LRP1 expression is normalized to the loading control. (**D**) Diagram showing the syndapin-2–mediated transcellular route and the intracellular degradation of LRP1.

The data in Fig. 1 showed that LRP1 is associated with several endocytic and trafficking elements, suggesting that the receptor is physiologically processed in endosomes and lysosomes, but it is also shuttled in tubular structures stabilized by syndapin-2. In addition, we demonstrated that transcytosis of LRP1 is driven by avidity and that fast shuttling across the BBB is associated with tubular structures. To further understand whether syndapin-2 is implicated into tubulation at BECs, we repeated the PLA assay between LRP1 and the endocytic proteins and syndapin-2, but this time, BECs were exposed to angiopep-2 peptide (*L* = 1) and A*_L_*-P formulations, *L* = 22 and *L* = 110. The data reported in Fig. 3A as a variation between the treated and untreated cells reveal how the avidity of the ligand for LRP1 affects the localization of the receptor within the cells. At an early incubation time (0.25 hour), the single peptide (*L* = 1) reduces the proximity events between LRP1 and clathrin by more than five times, while prolonged incubation promotes the association of the receptor with the late endosome marker Rab7. The two A*_L_*-P formulations have a more marked effect. Incubation with *L* = 22 prevents the interaction of LRP1 with all the endolysosomal compartments at any time point, with Rab5 showing the most notable decrease. The presence of *L* = 22 at 0.25 hour also increases the interaction of the receptor with actin, tubulin, and clathrin. On the other hand, incubation with *L* = 110 increases LRP1 interaction with both Rab7 and Rab11 in the short term, while it constantly reduces the association with syndapin-2. At later time points, incubation with *L* = 110 also decreases the proximity between LRP1 and tubulin or clathrin. Note that oscillations of the values between −5 and +5 were considered as physiological fluctuations. Our data suggest two trends: one is the association of LRP1 with syndapin-2 and one is with Rab5. We then plotted the ratio of the relative interactions between LRP1/Rab5 and LRP1/syndapin-2 (Rab5/syndapin-2) as a function of incubation time and ligand number (Fig. 3B). While angiopep-2 peptide does not alter the Rab5/syndapin-2 ratio, both *L* = 22 and *L* = 110 do but with opposite trends. *L* = 22 pushes the interaction of LRP1 toward syndapin-2 for all time points, while *L* = 110 biases LRP1 toward the endosomal protein Rab5. As we expected that LRP1 association with endolysosomal markers should result in its degradation, we assessed its levels of expression over time following incubation with *L* = 1, *L* = 22, and *L* = 110 (Fig. 3C). The WB results show that LRP1 is unaltered after up to 1-hour incubation with angiopep-2 and *L* = 22. In contrast, exposure to *L* = 110 results in a fast reduction of LRP1 expression, which then recovers to physiological levels after 2 hours of incubation. We observed a twofold increase in LRP1 expression after 2 hours of incubation with *L* = 22. Overall, both PLA and WB analyses suggest that LRP1 can follow two different intracellular pathways across BECs and their schematics are shown in Fig. 3D. One pathway is mediated by syndapin-2, β-actin, and, possibly, clathrin. Here, LRP1 shuttles across tubular carriers from apical to basal and vice versa, avoiding endolysosomal degradation and sorting. The other pathway is a conventional endocytosis where LRP1 enters the cells and gets trafficked to endosomes and lysosomes where it is degraded. On the basis of our findings, these pathways are driven by cargo avidity: Intermediate ligand numbers push more to the syndapin-2 pathway associated with tubular deformations, while the higher number of ligands and avidity pushes the cargo more toward endosomal sorting.

### Tubular transcytosis mechanism

To further shed light on the novel shuttling mechanism, we used a combination of qualitative and quantitative confocal microscopy in conjunction with antibodies and small-molecule inhibitors against proteins of interest. First, we coincubated the A_22_-P with the free peptide to provide an insight on whether transcytosis is more efficient when angiopep-2 is alone or when attached to the POs. Quantification of fluorescence is shown in fig. S6A. After 10 min of coincubation, A_22_-P fluorescence is of similar intensity of angiopep-2 and much lower than that of A_22_-P after 10 min with no competing ligand. Free peptide fluorescence remains similar to levels without competition. Such results show that angiopep-2 and A_22_-P compete for LRP1 binding and endocytosis, as expected, but also that the free peptide inhibits PO internalization more than vice versa. When coincubated, the intensities of A_22_-P and angiopep-2 are both markedly higher at 60 min compared to when added without competition. However, competition for A_22_-P shows a biphasic shift in behavior compared to the A_22_-P only control: decreased endocytosis at 10 min and increased intracellular residence, i.e., decreased exocytosis at 60 min. The biased inhibition of A_22_-P transcytosis rather than angiopep-2 may be due to more rapid or efficient endocytosis, intracellular trafficking, and exocytosis pathway occurring for A_22_-P than for angiopep-2.

We subsequently studied the mechanisms of endo- and exocytosis of Cy5-labeled A_22_-P during transcytosis. Confocal studies suggested that clathrin, but not caveolin, is involved in the mechanism of internalization of A_22_-P (fig. S6, B and C). High-magnification confocal images in fig. S6B demonstrate that A_22_-P fluorescence is closely associated with clathrin after 60 min of incubation. However, these data are qualitative and are thus only an indication that clathrin is involved in transcytosis of A_22_-P. We performed similar experiments to evaluate the association of Cy5-labeled A_22_-P with caveolin-1, and as shown in fig. S6C, a partial overlap was observed initially at 10 min of incubation. However, 3D *z*-stack projections in fig. S6D display no apparent colocalization at 10 min. A few cytoplasmic puncta with fluorescence overlap were observed at 60 min. However, *r* values for A_22_-P and caveolin-1 remained low along the time with *r* = 0.2 and −0.02 at 10 and 60 min, respectively. Overall, these findings fail to show a role for caveolae as essential structures for apical and basal transcytosis, particularly, as a higher colocalization would be anticipated at 10 min when the majority of transcytosis is occurring. Cytoskeletal motor proteins can quickly transport cargo from one side of a cell to another and were therefore of particular interest for their potential involvement in transcytosis. We thus investigated the role of actin in BEC transcytosis by colocalization of Cy5-labeled A_22_-P with phalloid-488 (an established marker for F-actin). Confocal images are displayed in fig. S6E, with a magnification of an area of interest (fig. S6E1), along with *r* values at 10, 30, and 60 min for A_22_-P and F-actin. The data suggest that actin has a role in transporting POs from the apical to basal membrane within the first few minutes of endocytosis. The time scale of BEC transcytosis and unconventional intracellular trafficking pathways prompted us to further explore the identity of intracellular transport vesicles as well as membrane deformation mediators in transcytosis. Small-molecule inhibitors of endocytosis or exocytosis were used in conjunction with live-cell imaging to obtain transwell *z* stacks. Incubation with dynasore, a cell-permeable inhibitor of dynamin, impaired transcytosis and caused Cy5-labeled A_22_-P to remain stuck on the BEC surface (fig. S6, F and G). These effects were reversible upon removal of the inhibitor, as the A_22_-P were visible both inside cells and in transwell membrane pores. Dynamin may, therefore, be a required cellular component of the internalization stage of transcytosis in BECs. In a separate experiment, *N*-ethylmaleimide (NEM) was used to inhibit NEM soluble factor (NSF) to inhibit exocytosis indirectly. A_22_-P remained aggregated on top of the cells after incubation for 60 min (fig. S6, F and G). Thus, NSF may participate not only in exocytosis of cargos once inside the cell but also in endocytosis. To further explore the role of NSF and soluble NSF attachment receptors (SNAREs) in transcytosis, a cell membrane cholesterol depletion method was used to disrupt lipid raft containing SNAREs (*49*). Cells were preincubated for 60 min with methyl-β-cyclodextrin (CD) added to either the apical or basal compartment of the transwell. A cholesterol quantification assay revealed a slight asymmetry in measured free cholesterol in the medium in the apical and basal compartments (fig. S7A). Depletion of cholesterol in the apical or basal membrane resulted in an approximately twofold or three- to fourfold increase in cholesterol released into the apical or basal side of the transwell, respectively (fig. S7B). Such an effect may be indicative of a stronger effect of cholesterol depletion on the basal membrane. Confocal images were acquired from Cy5-labeled A_22_-P incubated for 60 min in BECs with CD added to the apical or basal side of the transwell (fig. S7C). Basal membrane cholesterol depletion showed an increase in intracellular A_22_-P after 60 min compared to untreated cells, which may be due to the ability of cells to do endocytose but not exocytose the cargo. Together, these findings suggest the involvement of dynamin and also NSF in the LRP1-mediated cargo internalization stage of BEC transcytosis. Depletion of cholesterol in the basal side of BECs inhibited exocytosis but not endocytosis, which may suggest a role for cholesterol in transcytosis. We next assessed whether the trafficking from apical to basal involves sorting into endosomes and acidification, as we already demonstrated (*30*). A_22_-P do not colocalize with endosome and lysosomes crossing the BECs without losing integrity. Here, we represent confocal images acquired from A_22_-P in BECs fixed and stained for Rab guanosine triphosphatases of endosomal organelles in fig. S7D. There was no colocalization between POs and any of the markers at any time investigated. Colocalization quantification (fig. S7E) indicated no association between A_22_-P and Rab5, Rab7, Rab11, and LAMP-1. On the contrary, *r* values displayed a negative trend implicating negative association, i.e., exclusion of A_22_-P from these organelles.

**Fig. 4:**
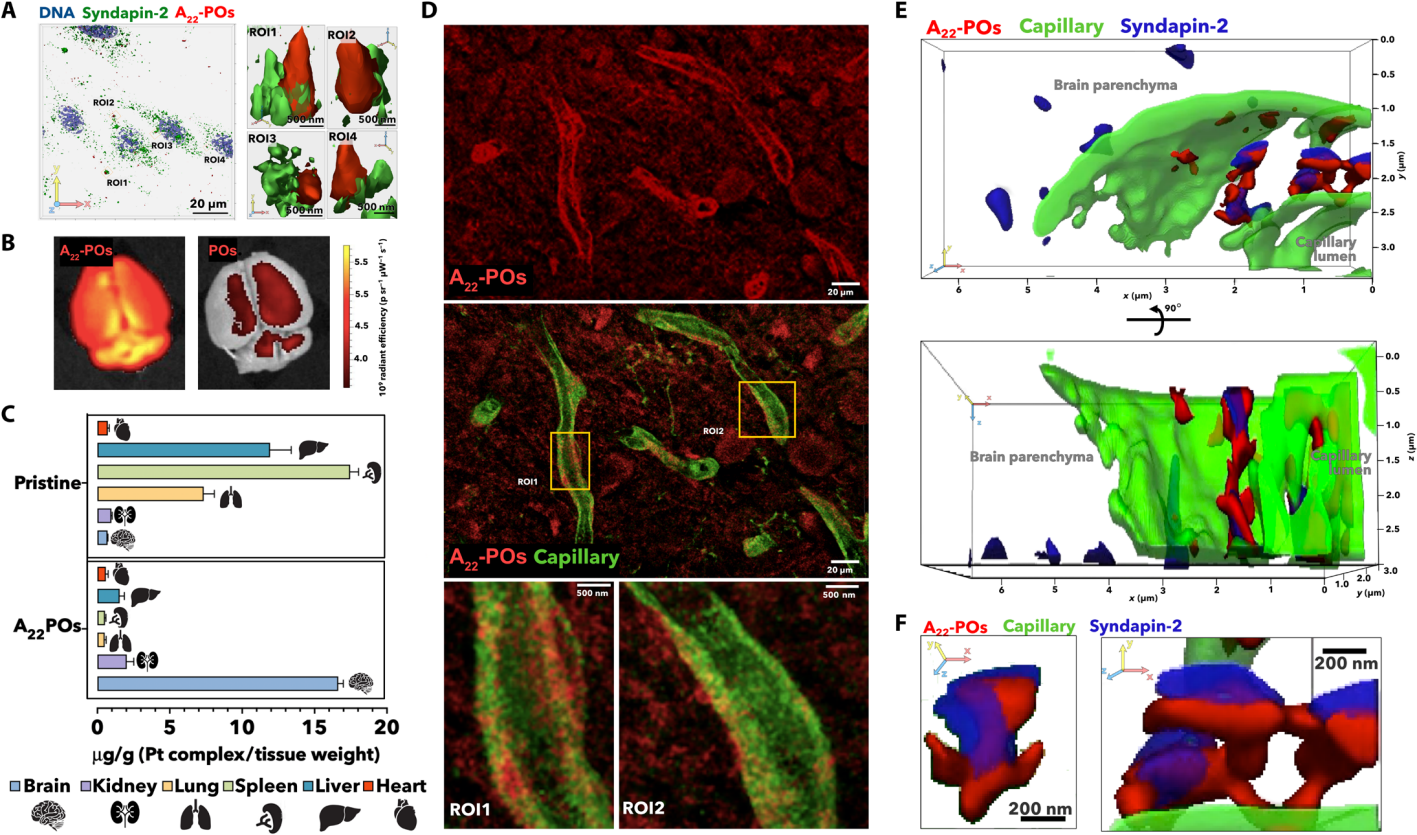
Syndapin-2–mediated transport. 3D rendering of confocal laser scanning micrographs of polarized BECs incubated with A_22_-P (red). (**A**) Cell nuclei were stained with DNA binding DAPI (blue), and syndapin-2 is shown in green (anti-IgG). (**B**) Fluorescence photograph of ex vivo whole mouse brains imaged 30 min after intravenous injection of A_22_-P and pristine POs loaded with PtA2. Pt tissue concentration in brain, kidney, lung, spleen, liver, and heart expressed as microgram per gram and measured by ICP-MS. (**C**) Tissues were collected 30 min after intravenous injection of A_22_-P and pristine POs both loaded with PtA2. STED micrographs of coronal brain sections showing the distribution of PtA2-loaded A_22_-P (red) 30 min after injection with capillary stained by lectin (green). (**D**) Two different regions of interests (ROIs) show the detail of the tubulation across the BECs. (**E**) 3D renderings as projections of STED micrographs of brain capillary (lectin in green) showing the detail of PtA2-loaded A_22_-P (red) and syndapin-2 (anti-IgG stained in blue). (**F**) Details of the tubule formed by the PtA2-loaded A_22_-P (red) surrounded by syndapin-2 (anti-IgG stained in blue).

Last, we confirmed the colocalization between A_22_-P and syndapin-2 in our in vitro BBB model. In Fig. 4A, 3D rendering of polarized BECs imaged 30 min after incubation with the Cy5-labeled A_22_-P (red) shows very effectively that A_22_-P cross the cell through tubular structures coated with syndapin-2 (in green). To further show the involvement of syndapin-2 on the transcytosis of A_22_-P, we modulated the expression of syndapin-2 on BECs and assessed the transport of A_22_-P across an in vitro BBB model (fig. S8). Specifically, we performed short hairpin RNA (shRNA) on bEnd3 to knock down syndapin-2, generating a stable cell line expressing significantly less syndapin-2, as confirmed by WB (fig. S8A). When cultured onto collagen-coated transwells, these syndapin-2 knockdown bEnd3 showed permeability *P*_4kDa_ = 25.6 and *P*_70kDa_ = 5.4 nm s^−1^, which are similar to the values obtained for bEnd3 transfected with a control shRNA (fig. S8B). We then assessed the transport of A_22_-P across BECs expressing different levels of syndapin-2. In fig. S8C, we observe a twofold decrease in the apparent permeability of A_22_-P from apical to basal side when compared to bEnd3 expressing normal endogenous levels of syndapin-2. These results further indicate the involvement of syndapin-2 in the transport across BECs. We complemented the colocalization of syndapin-2 and A_22_-P with animal studies where we injected either A_22_-P or pristine POs loaded with PtA2. In Fig. 4B, the ex vivo fluorescent photographs of whole brains extracted from healthy mice 30 min after injection show the effective delivery of the dye by functionalized POs. PtA2 has unique fluorescence characteristics with a wide Stoke shift and extremely bright emission, allowing us to visualize the PO penetration with high sensitivity. The metallic nature of the dye allows quantification of its biodistribution by ICP-MS. The graph in Fig. 4C shows an extremely effective delivery of the dye into the brain with a staggering brain/liver ratio of about 8.2 opposite to the pristine POs, where the majority of the dye is found in liver and spleen. Such a high concentration of dye allows us to visualize A_22_-P penetration in the brain capillary by TEM (fig. S9A) and STED. The histology in Fig. 4D demonstrates that A_22_-P cross the brain endothelium (stained with lectin in green) via the formation of tubules as shown in regions of interest 1 and 2. We then imaged brain sections collecting 30 optical slides, and the corresponding 3D renderings are shown in Fig. 4E, where the A_22_-P loaded with PtA2 (red) are imaged alongside the capillary walls (green) and syndapin-2 (blue) with improved spatial resolution. The rendering showed very well that A_22_-P colocalize into tubular structures coated by the F-BAR protein syndapin-2, with dimensions in agreement of what we observed in vitro and by the simulations.

### Tubular transcytosis dynamics

**Fig. 5:**
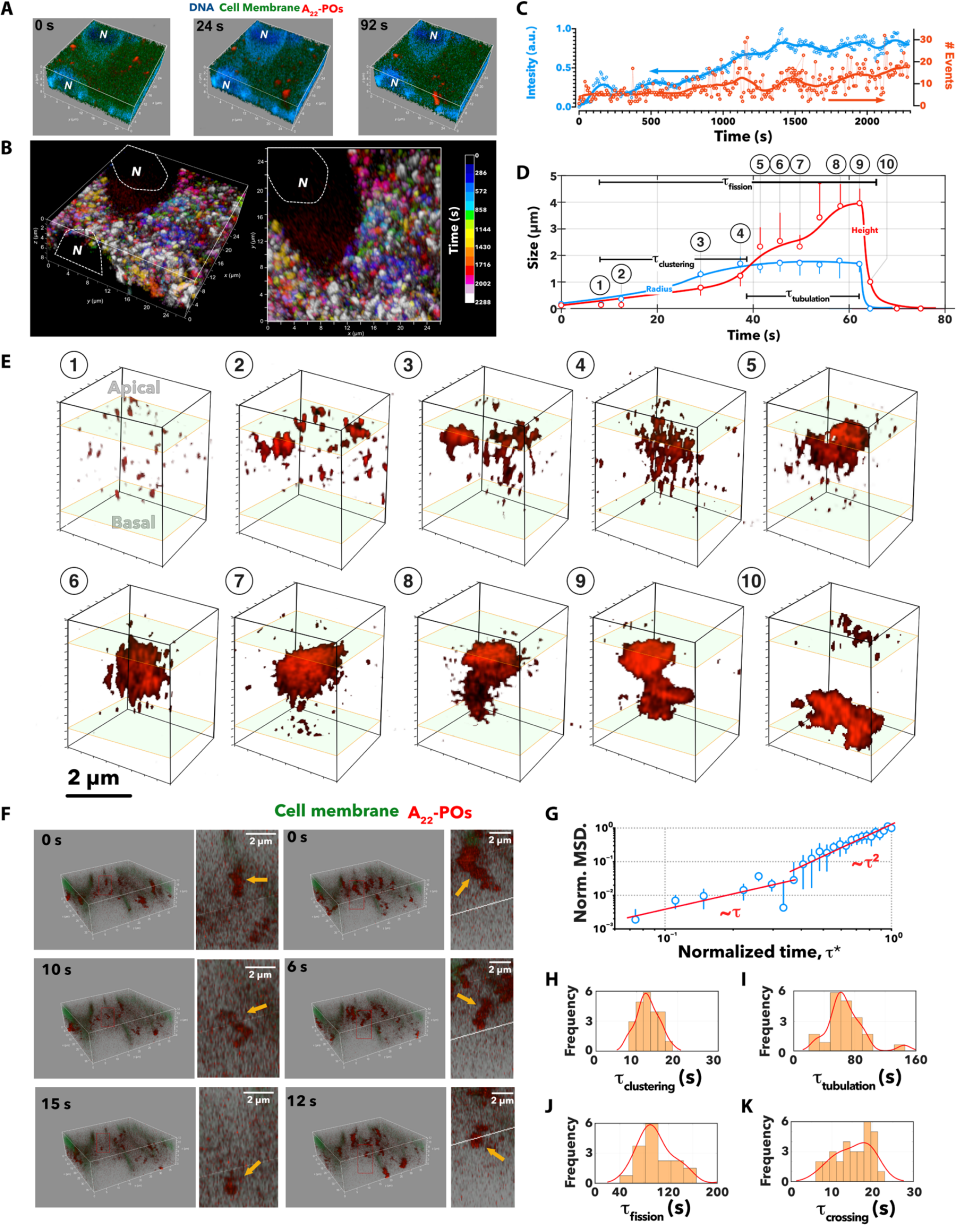
4D microscopy of transcytosis. (**A**) 3D rendering at three different times extracted from 4D (*xyzt*) live imaging of BECs stained for cellular membrane CellMask (green) and nuclei (blue) and incubated with Cy5-labeled A_22_-P (red). (**B**) 3D renderings of the same cell, with each event color-coded by its occurrence within periods of 144 s. (**C**) Graphs showing the red channel fluorescence intensity and the number of events (threshold in the red channel) as a function of time and zeta-averaged across the full cell thickness. a.u., arbitrary units. (**D**) Each event radius and length is monitored over time, and the average values across 20 events are plotted as a function of time. (**E**) The corresponding 3D renderings of the single events show an evolution from few puncta to large clusters, to membrane-bound tubulations, to tubular carriers. (**F**) Two sequences of 3D renderings extracted by fast 4D videos of the tubular carriers filled up with Cy5-labeled A_22_-P (red) crossing from one side to other BECs; note the cell membrane is stained by CellMask (green). (**G**) Normalized MSD as a function of normalized time τ*=tτcrossing, where τ_crossing_ is the time each event takes to fully cross from apical to basal and vice versa. Distribution of τ_clustering_ (**H**), τ_tubulation_ (**I**), and τ_fission_ (**J**) measured from the graph in (D) and τ_crossing_ (**K**) measured from the graph in (G).

As described above, A_22_-P formulation is extremely effective in crossing the BBB and thus makes the ideal tool to study the transcytosis dynamics. In fig. S9A, we show optically reconstructed sections of fixed polarized BECs incubated at different times with A_22_-P and stained for claudin-5. A_22_-P interact very quickly with the cells, and within 10 min, we observe POs crossing into the porous membrane. As shown by the costaining with claudin-5, A_22_-P seems to concentrate through the cells, and almost no fluorescence is observed in the tight junctions, indicating that A_22_-P diffusion across endothelial cells is transcellular rather than paracellular. We then measured the average fluorescence across apical to basal direction at different time points and disclosed that the transport dynamics are fast and occur within the first hour (fig. S9B). We thus stained polarized BECs with both DNA [4′,6-diamidino-2-phenylindole (DAPI)] and membrane stain (CellMask) and imaged the binding and crossing of A_22_-P using real-time 4D (*xyzt*) live-cell imaging. At first, we collected 3D sections every 8.2 s to generate movie S1. In Fig. 5A, three sequential 3D renderings show that A_22_-P (red) interact with the cell membrane forming several clusters that rapidly evolve into tubular endocytic events. Over the duration of the movie (40 min), we counted a total of 250 complete events, which are shown in the 3D rendering in Fig. 5B color-coded according to their occurrence. The time-sampled rendering shows that there are no events over the nuclear and perinuclear endoplasmic reticulum, and most of them are stochastically distributed over the remaining cell surface with a considerable level of overlapping of events occurring at different times but in the same spot. A_22_-P fluorescence intensity and the number of events were analyzed as a function of time and zeta-averaged across the full cell thickness (Fig. 5C). Furthermore, each event was analyzed by measuring its radius *r*_e_ and height *h*_e_ as a function of time (Fig. 5D), and one exemplary event is shown in its 3D renderings in Fig. 5E. In early stages, a very few puncta, with size below the capability of confocal laser scanning microscopy, were visible on the cell surface. These puncta assembled forming clusters with areas ranging from a few square micrometers to 70 μm^2^, with an average of about 25 μm^2^. The lateral growth of the clusters stops while they mature into tubular structures, with a height up to 6 μm and an average radius of 2.52 μm. After reaching an average aspect ratio of 1.8, the tubules disappear from the field of view faster than our time resolution, allowing us to partially capture the crossing. To capture the crossing dynamics, we also run experiments at faster acquisition times, where each optical section is collected in 2 s. Movie S2 and the snapshots in Fig. 5F show a strong interaction between A_22_-P and the BECs again. While the imaging quality is inevitably compromised, making the visualization of small early-stage events challenging, we can capture the dynamics of large tubular structures moving from one side to the other. We can thus measure the time each tubule takes to go across, defined here as crossing time τ_crossing_. In Fig. 5G, we plot the normalized mean square displacement (MSD) averaged across 51 events as a function of the normalized time calculated as τ*=tτcrossing. The data show that the tubule MSD follows a two-regime trend: At early stages, it is linear with time indicative of diffusional processes, while at later stages, the trends become parabolic typical of a ballistic process. The two dynamic analyses together allow us to identify four stages of transcytosis: (i) clustering, (ii) tubulation, (iii) fission, and (iv) crossing. At early stages, the cargo is sorted and clustered in an average of 13.5 s, where clustering time (τ_clustering_) was measured when *r*_e_ ≥ *h*_e_ (Fig. 5H). Then, the tubulation starts and it occurs in between 20 and 160 s, where τ_tubulation_ is measured when *r*_e_ < *h*_e_ (Fig. 5I). This time variance is the reflection of two kinetic processes that we observed. In some cases, the arrest of lateral growth of the clusters leads to an immediate tubulation, while in other instances, the tubules roam on the cellular surface for several seconds before disappearing. Next, the tube fission moves from one side of the cell to the other with a mean value of 110 s, where fission time (τ_fission_) is calculated as τ_clustering_ + τ_tubulation_ (Fig. 5J). The crossing time defined as above is less spread, with a mean value of about 15 s (Fig. 5K).

**Fig. 6:**
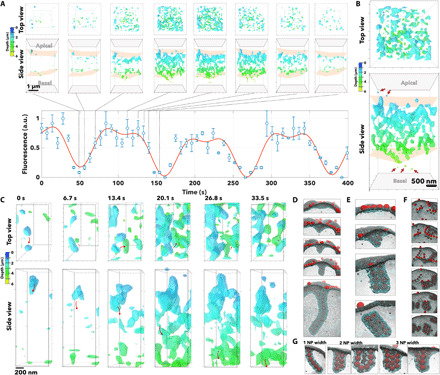
Super-resolution imaging of the tubular carriers. (**A**) 3D renderings shown as top and side views color-coded as a function of the depth (i.e., the distance from apical to basal) of optical sections of BECs incubated with PtA2-loaded A_22_-P. The 3D rendering was captured at a different time, and the normalized fluorescence measured across each section is plotted as a function of time. (**B**) The 3D rendering at 120 s is shown enlarged as top and side views and the arrows point at single A_22_-P particles, while the structure emerged as a network of tubules. (**C**) Close-up detail of top and side view of the same 3D rendering shows the evolution of the single tubule from apical to basal membrane showing the different stages of tubulation, fission from the apical membrane, and transport and fusion to the basal membrane. The same tubulations were observed in MD simulations. Here, the anisotropic growth of the membrane emerges from the collapse of a tubular aggregate of a particle on the surface. The membrane buckling can occur in different ways depending on the cluster size of assembled nanoparticles, leading to the formation of tubules (**D**), short tubes (**E**), or multiple assembly tubules (**F**). (**G**) The final tubule can thus be one, two, or three nanoparticles (NP) thick.

We repeated the live-cell imaging of polarized BECs using, this time, STED microscopy and A_22_-P encapsulating the PtA2 to achieve a spatial resolution close to 40 nm. BECs incubated with PtA2–A_22_-P were monitored in STED mode every 6.7 s for about 6 min. In Fig. 6A and movies S3 to S5, we show the corresponding 3D renderings color-coded in terms of depth. We also plotted the *z*-averaged fluorescence as a function of time, showing a remarkable periodicity in the formation of transcytotic events with a similar period to those we observed in Fig. 5C. STED resolution allows us to resolve the single nanoparticles, as shown in detail in Fig. 6B. The most notable revelation is that the transcytotic event emerges as an assembly of many tubular units each having an average diameter of about 100 ± 20 nm and a length varying from few hundreds of nanometers to few micrometers. In Fig. 6C, we show the evolution of these tubes from a few nanoparticles wide to large interconnected networks. An interesting observation is that the events appear very symmetrical, starting from both apical and basal sides, and growing until they are connected via a network of discontinuous tubes. It is important to point out that in both conventional and super-resolution imaging, we observed a dissociative tubule formation with fission preceding fusion, and hence, we did not observe a single tubule spanning from apical to basal. As we show in Fig. 2F, the intermediate binding energy results in nanoparticles within tubular aggregations, which are then internalized into the membrane (Fig. 6D). Typically, the nanoparticles first formed short linear surface aggregates that acted as nucleation seeds and grew in length. At some point, the linear aggregate buckles into the membrane, forming a membrane-bound tubular aggregation wrapped in an envelope of receptor beads that protruded into the inside of the membrane. The membrane-bound aggregations would then be internalized, forming a separate vesicle inside the membrane. Figure 6D shows that once the membrane buckles and starts deforming, it acts as a sink for the other nanoparticles bound to the membrane, resulting in the formation of a relatively long tubule. In most cases, the tubes undergo fission and sever from the membrane, as shown in Fig. 6E. The membrane “sinks” can attract more than one linear membrane-bound aggregate, resulting in the formation of tubules that are two or more nanoparticles thick (Fig. 6F). Immediately after their formation, but preceding any further growth or interactions, the membrane-bound tubular aggregates could be grouped into three different morphologies (Fig. 6G), depending on whether they are one, two, or three nanoparticles wide. Variations in the exact structure of the membrane-bound tubular aggregates were often seen, with aggregates of widths above three nanoparticles occasionally observed to arise from the merging of smaller aggregates. On one occasion, with an increased number of nanoparticles (200 instead of the usual 105), we observed a particularly large tubular structure (Fig. 6D). It was speculated that on a larger computer model, more aggregates could cooperatively take part in tubular growth interactions to give rise to even larger tubular aggregations of nanoparticles. For membrane-bound and internalized aggregates, the wrapping and deformation of the membrane occurs either by tight packing of the nanoparticles or via the formation of *U* structures, as can be seen in Fig. 6G. Astonishingly, the morphological structures observed in these simulations replicate very closely the tubular structures observed in both conventional and STED confocal microscopy, suggesting that the binding energy controls the membrane deformations and consequently how the cell processes vesicular carriers.

Together, our findings show a detailed picture of how avidity of the cargo affects trafficking of LRP1 across BECs and unravel a unique mechanism of tubular transcytosis mediated by syndapin-2 for fast shuttling of therapeutics across the BBB. We first demonstrate that LRP1 is processed by both endolysosomal compartments as well as by tubular carriers that very likely distribute it between apical and basal membranes (Fig. 1). We show that these tubular carriers are associated with the F-BAR domain syndapin-2 protein, which probably functions as a structural stabilizer. By studying a model cargo targeting LRP1, we show that BECs sort the cargo depending on avidity (Fig. 2), and this controls trafficking at both the binding/unbinding and membrane deformation. We observe that such a bias extends to intracellular trafficking. At high binding energy, the single cargo is internalized via a conventional endocytic pathway leading to lysosomal sorting and degradation, while the mid binding energy (Goldilocks avidity) leads to a unique pathway controlled by tubular carriers. These tubular carriers resemble morphologies previously reported, such as TEC (*24*, *25*) and VVOs (*26*). We thus show that avidity enables high efficiency of transport across BBB and becomes a discriminant for nutrients required for the brain or for endothelial cells themselves. Such a bias, in turn, alters the targeted receptor expression on BECs with the collective endocytosis and nondegradative tubular carrier pathway, leading to LRP1 up-regulation, while the single endocytic internalization and endolysosomal pathway leads to down-regulation (Fig. 3). The result, as demonstrated above, is that in the former the cargo is shuttled across efficiently, while in the latter it is degraded and possibly consumed by the same endothelial cell. Moreover, the ability to image the tubular carriers, by using the avidity optimized synthetic carrier A_22_-P, allowed us to reconstruct their morphology with an unprecedented resolution both in vitro and in vivo, showing the critical role of syndapin-2 in stabilizing them (Fig. 4) and confirming previous TEM reconstructions (*25*). We report here the dynamic of tubular formation and occurrence as well as their distribution over the cell surface (Fig. 5). To this extent, the use of super-resolution imaging in combination with MD simulation presents a critical role of the cargo in templating the tubular formation and dynamics (Fig. 6). In conclusion, we shed light on BEC transcytosis using LRP1 as the main actor, but considering that Goldilocks avidity effect was also reported for transferrin receptor (*20*–*22*) and GLUT1 (*23*), our findings might suggest similar mechanisms. We demonstrate that the fast tubular structures are associated with syndapin-2, providing the first evidence of the involvement of a BAR protein in transcytosis at the BBB. Nevertheless, ultimately, we report here a clear path to the brain, which, once optimized, allows efficient delivery of therapeutics to the brain.

## MATERIALS AND METHODS

### Materials

Polymers were obtained through atom transfer radical polymerization (ATRP) synthesis as previously reported (*30*). bEnd3 (CRL-2299), Dulbecco’s modified Eagle’s medium (DMEM), and FluoroBrite DMEM were obtained from the American Type Culture Collection. Fetal bovine serum (FBS), penicillin/streptomycin, phosphate-buffered saline (PBS; pH 7.4), 0.25% trypsin-EDTA, rat tail collagen I, 3- to 5-kDa fluorescein isothiocyanate (FITC)–dextran, and 65- to 85-kDa tetramethylrhodamine isothiocyanate (TRITC)–dextran were obtained from Sigma-Aldrich. Transwell permeable polyester membranes (400 nm pore, 1.12 cm^−2^) were obtained from Corning Inc. EVOM2 Epithelial Voltohmmeter with STX3 electrodes was purchased from Word Precision Instruments. Paraformaldehyde (PFA), Triton X-100, normal horse serum, FITC-conjugated lectin, PLA probe anti-rabbit PLUS, PLA probe anti-mouse MINUS, Duolink detection reagent orange, radioimmunoprecipitation assay (RIPA) buffer, Tween 20, dextran (60 to 76 kDa), dynasore, NEM, methyl-β-CD, bovine serum albumin (BSA), and cholesterol quantification kit were also obtained from Sigma-Aldrich. Vectashield Mounting Media was obtained from Vector Labs. Protease inhibitors, BCA protein assay kit, and Laemmli sample buffer (4×) were purchased from Bio-Rad. Angiopep-2 was obtained from GenScript. Puromycin dihydrochloride, gentamicin, CellMask Deep Red plasma membrane, DAPI, and Leica standard immersion oil were obtained from Thermo Fisher Scientific. All antibodies used are listed above. Polybrene, syndapin-2 shRNA, and control shRNA lentiviral particles were purchased from Santa Cruz Biotechnology.

### Animal experiments

All animal studies were carried out according to the guidelines of ARRIVE (Animal Research: Reporting of In Vivo Experiments) under license from the UK Home Office (Scientific Procedures Act 1986) and approved by the University College London ethical review committee. Other set of animal experiments was carried out according to the national regulations and approved by the animal experiments ethical committee of School of Pharmaceutical Sciences and School of Chinese Medicine, Southwest University. In all experiments, animals were housed in a controlled temperature room with regular alternating cycles of light and darkness.

### Cell culture

Mouse BECs bEnd3 were used between passages 20 and 30. bEnd3 were grown in DMEM supplemented with 10% (v/v) FBS and penicillin (100 IU ml^−1^)/streptomycin (100 mg ml^−1^). Cells were maintained at 37^∘^C in an atmosphere of 5% CO_2_. For subculture, bEnd3 were washed twice with PBS, incubated with 0.25% trypsin-EDTA for 3 min, centrifuged, and resuspended in fresh medium. The medium was changed every 2 to 3 days.

### In vitro BBB model

To form a polarized confluent BEC monolayer, bEnd3 were seeded at a density of 25,000 cells cm^−2^ in collagen-coated polyester membranes. bEnd3 were grown for 3 days in complete DMEM medium containing 10% (v/v) FBS and then switched to serum-free medium in the basal side of the transwell membrane for another 3 days. On day 6, TEER was measured using an EVOM2 and the expression of PECAM, claudin-5, and ZO-1 was assessed by immunofluorescence. Dextran (3 to 5 kDa and 65 to 85 kDa) permeability across the endothelial monolayers was also assessed. A detailed description of the permeability assays can be found in the Supplementary Materials.

### Polymersome preparation

A description of PO preparation and characterization via DLS and TEM can be found in the Supplementary Materials.

### Immunofluorescence

Polarized bEnd3 monolayers either untreated or treated with A_22_-P (500 μg ml^−1^) were washed twice with PBS, fixed in 4% (w/v) PFA for 15 min, permeabilized with 0.1% (w/v) Triton X-100 in PBS for 10 min, and incubated with 5% (w/v) BSA in PBS for 1 hour at room temperature. Afterward, cell monolayers were incubated with primary antibodies diluted in 1% (w/v) BSA and 0.01% (w/v) Triton X-100 in PBS overnight at 4°C, followed by washing with PBS and incubation with the corresponding secondary antibodies for 2 hours at room temperature. Nuclei were counterstained by incubation with DAPI for 10 min. Transwell membranes were excised using a scalpel and mounted on coverslips with Vectashield Mounting Media. Coronal brain sections were obtained from adult C57BL/6J (4 months old) mice. Briefly, brain sections were incubated in 20% (v/v) normal horse serum in PBS containing 0.3% (w/v) Triton X-100 for 2 hours at room temperature under gentle agitation followed by incubation with primary antibody anti–syndapin-2 overnight at 4^∘^C. Sections were washed with PBS, incubated with the corresponding secondary antibody and FITC-conjugated lectin (1:200) for 2 hours, and washed with PBS. Brain sections were mounted on glass slides in Vectashield Mounting Media. A list of antibodies can be found in the Supplementary Materials.

### Western blot

Polarized bEnd3, either untreated or treated with angiopep-2 (1.75 nM), A_22_-P, or A_110_-P (500 μg ml^−1^) for 0.25, 1, and 2 hours, were washed twice with PBS, and RIPA buffer containing protease inhibitors (1:50) was added directly to the membranes and left on ice for 1 hour. Cells were collected and centrifuged, and the supernatant was collected for WB analysis. Protein levels in the cell lysates were determined using the BCA Protein Assay Kit. Lysates were mixed with Laemmli sample buffer, and proteins (10 μg) were separated on 10% SDS polyacrylamide gels and transferred to polyvinylidene difluoride membranes. Membranes were blocked with 5% (w/v) nonfat milk in tris-buffered saline (TBS) containing 0.1% (w/v) Tween 20 (TBS-T) for 1 hour and then incubated with a rabbit monoclonal antibody to LRP1 overnight at 4^∘^C. After washing with TBS-T, the membranes were incubated with a secondary antibody for 2 hours at room temperature and imaged using Odyssey CLx (LI-COR Biosciences). The membranes were further probed for glyceraldehyde-3-phosphate dehydrogenase (GAPDH) as a loading control.

### Proximity ligation assay

Polarized bEnd3 [untreated or treated with angiopep-2 (1.75 nM), A_22_-P, or A_110_-P (500 μg ml^−1^) for 0.25, 1, and 2 hours] were washed twice with PBS, fixed in 4% (w/v) PFA in PBS for 15 min, and permeabilized with 0.1% (w/v) Triton X-100 in PBS for 10 min. For the PLA assay, the Duolink probes and detection reagents were used according to the supplier’s instructions. Briefly, monolayers were incubated with Duolink blocking solution for 1 hour at 37^∘^C and then incubated with two antibodies targeting the proteins of interest (one being LRP1 and the other one of the proteins relevant for transcytosis) overnight at 4^∘^C. Following incubation with primary antibodies, cells were incubated with the Duolink PLA probes (anti-rabbit and anti-mouse) for 1 hour at 37^∘^C, washed, and incubated with Duolink ligase and polymerase for 30 and 100 min, respectively. Nuclei were stained by adding DAPI for 10 min. Membranes were mounted in glass coverslips using Vectashield Mounting Media. A list of antibodies can be found in the Supplementary Materials.

PLA data were quantitatively analyzed using a Python script based on Trackpy modified for identification of particles with high polydispersity in the direction of objective translation (*z*). To each optical slice in the *z*-stack, a Gaussian filter was applied to remove short-wavelength detector noise and a low-pass rolling average filter was applied to remove large-scale features due to channel cross-talk. Local maxima were then identified, and maxima in the *z* direction corresponding to the same particle were grouped by hierarchical clustering using the nearest point algorithm as implemented in SciPy (*50*). For quantitative fluorescence measurements, total puncta intensities were normalised by voxel volume to account for variation in photon dose per unit volume as a function of imaging resolution.

### Permeability assays

To assess permeability across polarized bEnd3, Cy7-labeled A*_L_*-P with *L* = 0, 22, 36, 56, 110, and 220 (100 μg ml^−1^) and FITC–angiopep-2 (10 μg ml^−1^) were added to the apical side of the transwell membrane and incubated at 37^∘^C. Samples were collected from the basal side, and fresh medium was added to replace the volume. Fluorescence intensity of the Cy7-labeled A*_L_*P or FITC–angiopep-2 was measured in black 96-well plates using a Spark multimode microplate reader (Tecan). Apparent permeability was calculated using Eq. 1.

### In vivo biodistribution of Cy7-labeled POs

Healthy adult C57BL/6J female mice were injected via tail vein with 100 μl of either Cy5- and Cy7-labeled A*_L_*-P, with *L* = 0, 22, or 110 at a concentration of 4 mg ml^−1^ or with free Cy5–angiopep-2. Within 2 hours from the administration, mice were anaesthetized with isoflurane and imaged using an IVIS (PerkinElmer). Animals were then perfused with 50 ml of PBS, and brains were collected, imaged again using IVIS, and then snap-frozen in liquid nitrogen. To quantify brain accumulation, the cerebellum was removed. Cerebrum was weighed; PBS was added and manually homogenized with the addition of 3 volumes of 30% (w/v) dextran (64 to 74 kDa). Then, samples were centrifuged at 7400*g* for 20 min, which results in the separation into fractions: capillary-depleted fraction (i.e., parenchyma), dextran, and capillary-enriched fraction (i.e., vessels). Parenchyma was resuspended and added to a black 96-well plate. Fluorescence of the POs was measured in a Spark multimode microplate reader. Sample fluorescence readings were normalized to the ones obtained from the mice injected with PBS. Positive control was POs spiked at a known concentration in the homogenates (*n* = 3). Normalized fluorescence readings were converted to PO mass, which was then converted into the percentage of ID per gram (% ID/g) of tissue.

### In vivo biodistribution of PtA2-loaded POs and histological analysis

A detailed description of the synthesis and characterization of PtA2 and preparation of PtA2-loaded POs can be found in the Supplementary Materials. For the in vivo biodistribution of PtA2-loaded POs, 4-week-old Kunming mouse were injected via tail vein with POs (200 μl at 1 mg ml^−1^). At given time points, mice were culled with an overdose of isoflurane and perfused with PBS, and the organs were fixed in a solution of 4% (w/v) PFA in PBS. The organs were further fixed with 4% (w/v) PFA at 4^∘^C for 24 hours and dehydrated in a solution of 30% (w/v) sucrose in PBS for more than 48 hours. Next, the organs were weighed and digested in 60% (v/v) nitric acid at room temperature for 24 hours. Each organ was diluted in ultrapure water to achieve a final volume of 10 ml containing 3% (v/v) nitric acid. The concentration of platinum in each organ was determined using an ICP-MS (Thermo Fisher Scientific). Alternatively, the perfused brains were extracted, and dura mater was removed and postfixed for 7 hours in 4% PFA at 4^∘^C. Then, the fixed brains were immersed in 20% (w/v) sucrose in PBS overnight at 4^∘^C for cryoprotection. Fixed brains were cut using a cryostat (Leica 1950) at 20 μm in the coronal plane and mounted onto glass slides. Sections were initially washed three times in PBS for 5 min at room temperature and preincubated for 1 hour in a blocking buffer consisting of 2% (v/v) goat serum and 1% (w/v) BSA in 0.1% (v/v) Triton X-100 in PBS (PBS-T). After washing with PBS, processed sections were incubated with primary antibodies diluted in PBS for 1 hour at room temperature. Sections were then washed four times in PBS for 5 min each and incubated for 2 hours at room temperature using the appropriate secondary antibodies. For blood vessel labeling, mice were initially injected intravenously with FITC-labeled lectins (200 μl of 500 μg ml^−1^) at 5 min before culling.

### Confocal microscopy and imaging

For live kinetics, bEnd3 monolayers were incubated with CellMask Deep Red plasma membrane staining for 30 min, rinsed with PBS three times, and immersed in a FluoroBrite DMEM medium supplemented with 10% (v/v) FBS and gentamicin (5 mg ml^−1^). Subsequently, Cy5-labeled A_22_-P were added (1 mg ml^−1^) into the apical side of the transwell and incubated for 1 to 2 hours at 37^∘^C in 95% air and 5% CO_2_. Images were acquired using a Leica TCS SP8 confocal microscope equipped with Diode 405, Argon, DPSS 561, and HeNe633 lasers. Images were acquired via sequential scan to reduce fluorophore bleed-through. For live-cell imaging, an incubator at 37^∘^C and 5% CO_2_ connected to the unit was used and allowed to stabilize for 1 hour before imaging. For live-cell 3D scanning, images were acquired with a 10× 0.3 numerical aperture (NA) objective in resonant scanning mode with a speed of 700 Hz and a resolution of 128 × 512 pixels. For fixed cell imaging, images were acquired with a 63× oil immersion objective at 400 Hz and 512 × 512 pixels. Leica SP8 AFS microscope software was used to generate 3D projections from *z* stacks. The same software was used for analysis of PO fluorescence in transwell *z* stacks and normalization of fluorescence intensity. ImageJ was used for analysis of fluorescence intensity in the *xy* plane. Colocalization analysis to obtain Pearson’s correlation coefficient *r* was done using the plug-in for colocalization on ImageJ.

### STED microscopy imaging

STED and fast STED super-resolution imaging experiments were done using a Leica DMi8 confocal microscope equipped with a Leica TCS SP8 STED-ONE unit. PtA2-loaded POs were excited with a 405-nm laser, and emission was collected at 550 to 580 nm with donut laser at 595 nm. Images were collected using HyD reflected light detectors with 2048 × 2048 pixels and 100× scanning speed. For fast STED imaging, a resonant model was applied to minimize the 3D scanning time. Transwell membranes were imaged with a 100× lens. 3D images were recorded after POs were added into the apical side (thickness, 8 μm; interval, 0.2 μm; time interval, 5.0 to 8.0 s). STED and fast STED micrographs were further processed using “deconvolution wizard” function from Huygens Professional software under authorized license. The area radii were estimated under 0.03 μm with exclusion of 200 absolute background values. Maximum iterations were 10 times, and a signal-to-noise ratio of 30 was applied, with quality threshold at 0.005. Other settings were the “optimized” iteration mode and the “automatic” brick layout.

### Statistics

The results are expressed as mean ± SD. Comparisons between groups were obtained by one-way analysis of variance (ANOVA) using Dunnett’s post hoc test in the comparison to a control or Tukey’s for multiple comparisons between groups in GraphPad Prism 7.03. Significance level of *P* < 0.05 was considered statistically significant.

## Supplementary Material

http://advances.sciencemag.org/cgi/content/full/6/48/eabc4397/DC1

Movie S1

Movie S2

Movie S3

Movie S4

Movie S5

Adobe PDF - abc4397_SM.pdf

On the shuttling acros the blood-brain barrier via tubule formation: Mechanism and cargo avidity bias

## SUPPLEMENTARY MATERIALS

Supplementary material for this article is available at http://advances.sciencemag.org/cgi/content/full/6/48/eabc4397/DC1

## Appendix

View/request a protocol for this paper from *Bio-protocol*.
