# Supplementary material for: On the shuttling across the blood-brain barrier via tubule formation: Mechanism and cargo avidity bias
Source: Sci Adv. 2020 Nov 27;6(48):eabc4397. doi: 10.1126/sciadv.abc4397 (PMC7695481; doi:10.1126/sciadv.abc4397)
Supplement: http://advances.sciencemag.org/cgi/content/full/6/48/eabc4397/DC1 [file supp_6_48_eabc4397__index.html]

Science Advances | Science AdvancesAAASSearchScience AdvancesMenu

## Supplementary Materials

# On the shuttling across the blood-brain barrier via tubule formation: Mechanism and cargo avidity bias

Xiaohe Tian, Diana M. Leite, Edoardo Scarpa, Sophie Nyberg, Gavin Fullstone, Joe Forth, Diana Matias, Azzurra Apriceno, Alessandro Poma, Aroa Duro-Castano, Manish Vuyyuru, Lena Harker-Kirschneck, Anđela Šarić, Zhongping Zhang, Pan Xiang, Bin Fang, Yupeng Tian, Lei Luo, Loris Rizzello, Giuseppe Battaglia

Download Supplement

**This PDF file includes:**

- Supplementary text
- Table S1
- Figs. S1 to S9
- Legends for movies S1 to S5
- References

**Other Supplementary Material for this manuscript includes the following:**

- Movie S1
- Movie S2
- Movie S3
- Movie S4
- Movie S5

**Files in this Data Supplement:**

- Adobe PDF - abc4397\_SM.pdf
- abc4397\_Movie\_S1.mov
- abc4397\_Movie\_S2.mp4
- abc4397\_Movie\_S3.mov
- abc4397\_Movie\_S4.mov
- abc4397\_Movie\_S5.mov
