# Supplementary material for: On the shuttling across the blood-brain barrier via tubule formation: Mechanism and cargo avidity bias
Source: Sci Adv. 2020 Nov 27;6(48):eabc4397. doi: 10.1126/sciadv.abc4397 (PMC7695481; doi:10.1126/sciadv.abc4397)
Supplement: http://advances.sciencemag.org/cgi/content/full/6/48/eabc4397/DC1 [file supp_6_48_eabc4397__1.pdf]

Science Advances
AAAS

advances.sciencemag.org/cgi/content/full/6/48/eabc4397/DC1

Supplementary Materials for

On the shuttling across the blood-brain barrier via tubule formation: Mechanism and cargo avidity bias

Xiaohe Tian, Diana M. Leite, Edoardo Scarpa, Sophie Nyberg, Gavin Fullstone, Joe Forth, Diana Matias, Azzurra Apriceno, Alessandro Poma, Aroa Duro-Castano, Manish Vuyyuru, Lena Harker-Kirschneck, Anđela Šarić, Zhongping Zhang, Pan Xiang, Bin Fang, Yupeng Tian, Lei Luo, Loris Rizzello, Giuseppe Battaglia*

*Corresponding author. Email: g.battaglia@ucl.ac.uk

Published 27 November 2020, *Sci. Adv.* **6**, eabc4397 (2020)
DOI: 10.1126/sciadv.abc4397

The PDF file includes:

Supplementary text
Table S1
Figs. S1 to S9
Legends for movies S1 to S5
References

Other Supplementary Material for this manuscript includes the following:

(available at advances.sciencemag.org/cgi/content/full/6/48/eabc4397/DC1)

Movies S1 to S5

Supplementary Materials

Characterisation of the *in vitro* BBB Model bEnd3 were seeded at a density of 25,000 cells cm^{-2} in collagen-coated polyester membranes. Cells were grown for 3 days in complete DMEM medium containing 10% (v/v) FBS, and then switched to serum-free media in the basal side of the transwell membrane for another 3 days. On day 6, expression of PECAM, claudin-5 and ZO-1 was assessed by immuno-fluorescence. Dextrans (3-5 and 65-85 kDa) permeability across the endothelial monolayers was also assessed. Briefly, FITC- and TRITC-dextrans (1 mg mL^{-1}) were added to the apical side of the transwell membrane containing media in the basal side. At specific time-points, a sample was collected from the basolateral side and fresh media was used to replace the volume. Fluorescence of FITC and TRITC was measured in black 96 well-plates using Spark Multimode Microplate Reader (Tecan). Apparent permeability was calculated according to **Equation 1** (**Fig.S1**).

Preparation and characterisation of POs Poly(ethyleneglycol) methyl ether methacrylate $(POEGMA)_{20}$-poly(2-(diisopropylamino)ethyl methacrylate) ($PDPA_{100}$) copolymer was prepared according to a previously published procedure [30]. Angiopep-2- and Cy5-/Cy7-labelled $POEGMA_{20}$-$PDPA_{100}$ were prepared according to established protocols [30]. Angiopep-2 (0-12%mol), Cy5- or Cy7-(10%mol)-functionalised POs were prepared either by pH or solvent-switch approach. Briefly, in a pH-switch, copolymer (20 mg) was dissolved in PBS at pH 2, followed by gradually increasing the pH to 6.0 using 0.5 M NaOH under magnetic stirring. At pH 6.0, angiopep-2-$POEGMA_{20}$-$PDPA_{100}$ was added to the copolymer solution and the pH was then increased to 7.4. In solvent-switch, copolymer (20 mg) was dissolved in tetrahydrofuran (THF), and to this polymer solution, PBS (pH 7.4) was added using a syringe pump at a flow rate of 2 μL min^{-1} under stirring at 40 °C. A final volume of PBS (2.3 mL) was added. Once the volume of PBS was added, POs were dialysed against PBS (pH 7.4). In both approaches, POs were purified *via* a gel permeation chromatography on a size-exclusion column containing Sepharose 4B. Dynamic scattering (DLS) was used to assess average size and polydispersity of the POs by using a Malvern Zetasizer Nano ZS. POs were imaged by transmission electron microscopy (TEM) to assess size, surface topology and morphology using a JEOL microscope with a 100 kV voltage tension (**Fig.S3**).

Synthesis and characterisation of Pt2A Initially, FA2 compound was synthesised. Briefly, ammonium acetate (7.71 g, 0.10 mol), 4-methylbenzaldehyde (1.52 g, 0.10 mol), 1-tetralone (2.89 g, 0.02 mol) and glacial acetic acid (40 mL) were added to a 100 mL round-bottom flask and stirred for 24 hours at room temperature to obtain a burgundy solution. At the end of the reaction (monitored by thin layer chromatography), the resulting product was poured into an excess of water. The obtained solid was filtered by suction an purified by column chromatography using petroleum ether and ethyl acetate (10:1) as eluent. A white solid (5.03 g) was then obtained and further purified by recrystallising in ethyl acetate (yield of 89%). ^{1}H-nuclear magnetic resonance (^{1}H-NMR) was performed (400 MHz, CD3CN) showing the following peaks δ (ppm): 8.51 (d,J=7.7 Hz, 1H), 7.42 (t,J)=7.7 Hz, 1H), 7.35 (t,J)=7.4 Hz, 1H), 7.28 (d,J)=7.4 Hz, 4H), 7.07 (d,J)=8.4 Hz, 6H), 6.84 (d,J)=8.2 Hz, 2H), 3.45 (q,J)=7.0 Hz, 2H), 2.91-2.82 (m, 4H), 1.21 (t,J)=7.0 Hz, 4H). High resolution mass spectrometry (HRMS) electrospray ionisation mass spectrometry (ESI-MS) was also carried out, in which theroretical m/z is 373.49 and the one obtained was 374.19 ([M+H] +).

Once the FA2 compound was obtained, PtA2 was then synthesised. Briefly, FA2 (0.53 g, 1.3 mmol) and potassium tetrachloroplatinate(II) (0.50 g, 1.2 mmol) were mixed in acetic acid (150 mL). The liquid suspension was heated to 400 K for 72 hours under nitrogen atmosphere. A yellow-green residue was obtained after filtration. The intermediate, without further purification, was dissolved in dimethyl sulfoxide (5 mL) and refluxed for 30 minutes. Then, the reaction solution was treated with deionised water (50 mL). A yellow powder was obtained after filtration and dried under vaccum conditions. The purified product was obtained by column chromatography (neutral alumina, hexane:ethyl acetate)c = 5:1) as a yellow powder. Yield was found to be 53%. Final product was characterised by ^{1}H-NMR (400 MHz, d6-DMSO) showing the distinctive peaks δ (ppm): 7.46 (t,J=8.1 Hz, 2H), 7.35 (d,J=7.8 Hz, 2H), 7.23 (d,J=8.0 Hz, 2H), 7.07 (dd,J=15.3, 8.0 Hz, 2H), 6.83 (dd,J=31.1, 7.5 Hz, 2H), 2.78 (t,J=7.6 Hz, 4H), 2.53 (d,J=5.0 Hz, 10H), 2.40 (s, 3H). Fourier-transform infrared spectroscopy (FTIR))was also performed (KBr, v, cm^{-1}) exhibiting the peaks: 3433, 3037, 2912, 1603, 1567, 1390, 1310, 1244, 1112, 1016, 765, 685, 450. HRMS (ESI-MS) was performed with a calculated m/z of 644.69 and the m/z found of 645.15 ([M+H] +) (**Fig.S4**).

Preparation of PtA2-loaded POs PtA2 (1 mg) and copolymer (10 mg) were individually dissolved into a mixture of methanol and dichloromethane (5 mL) in separate vials. Both solutions were then mixed uniformly into a 10 mL flat-bottom glass bottle, and rotaevaporated at 40 °C to obtain a thin film. The resulting film

was hydrated with PBS (5 mL) and left to stir at room temperature for at least 2 weeks. Morphology and PtA2 encapsulation were evaluated by TEM and fluorescence spectrophotometry, respectively.

Transcytosis model Nanoparticles were randomly seeded within the aqueous phase in the starting states. Nanoparticles in the aqueous phase moved according to Brownian motion with a time step of 0.00005 seconds. Dynamic viscosity was given as 0.00078 Pa $^{-1}$, which corresponds to the viscosity of the cell medium DMEM at a standard temperature of 37 $^{\circ}$ (310 K) [51]. The boundaries of the transwell, the top of the aqueous phase and the edge of the cell layer were treated as reflective boundaries. The characteristic length for LRP1-angiopep-2 was estimated to be 6.5 nm based on the molecular weight of LRP1β (the extracellular chains that forms part of the LRP1 heterodimer) using the methods of Erickson [52, 53]. Nanoparticles in close enough proximity to the top of the cell layer are able to form bonds with the cell. Temporary ligand agents were created randomly outside of these nanoparticles to the required density as this method would be computationally less expensive than adding rotational diffusion and updating the positions of the ligands each iteration. Moreover, for the time step used for receptor binding (0.01 seconds) and the radius of the nanoparticles, the contact surface is effectively randomised each iteration by rotational diffusion so that recreation of the ligands would not adversely effect the robustness of the model compared to incorporating full rotational diffusion. Therefore, this method was adapted to give a simulation of binding and unbinding across discrete steps. Decuzzi and Ferrari investigated the binding of nanoparticles in a static linear flow, they gave the probability of a nanoparticle adhering as:

$$P_a \simeq R_D L_D k_a^0 A_C \exp(-\frac{B_{RL} F_{RL}}{K_B T}) \quad (1)$$

R_D is the receptor density on the cell surface, k_a^0 is the association constant at zero load per receptor-ligand pair and A_C is the contact area between nanoparticle and cell surface.

The interfacial contact surface area, the receptor density and the ligand density dictate the number of bonds between the particle and a cell. The target cell determines the receptor density whilst ligand density is an adaptable property of the nanoparticles. The interfacial surface area is the surface area of the nanoparticle that is within a set binding distance from the cell. The characteristic length of the receptor-ligand bond. We then build an agent-based model of nanoparticle-cell binding unit based on the transwell *in vitro* model of the BBB to investigate how adapting the ligand density and nanoparticle size can alter transcytosis efficiency (**Fig.S5**).

Molecular dynamics simulations A meshless solvent-free coarse-grained (CG) membrane model was coupled with molecular dynamics (MD) simulations to capture membrane topological changes and nanoparticle aggregation dynamics at the required length and time scales. In the model, the membrane was discretised into beads that each represent a CG membrane surface patch (**Fig.S5**). Different beads were used to represent a membrane surface patch with no receptors ('inert' bead) and a membrane surface patch with receptors ('receptor' bead). The beads self-assembled into a membrane and replicated biologically relevant properties using a soft-core pairwise inter-particle potential [53]. An equilibrated spherical membrane of 20162 membrane beads of diameter 1σ (where$\sigma$is the MD unit of length) was used in the simulations. Typically, nanoparticles were represented as beads of diameter 4σ in the model. 105 nanoparticles were randomly distributed in a spherical shell surrounding the membrane with a radius R_M + 6σ, where $R_M$is the undeformed average radius of the membrane. A minimum distance of 5σ between the centers of the nanoparticles was enforced in this initial distribution. The repulsive branch of a 12-6 Lennard-Jones potential was used for the volume exclusion of the nanoparticles. The nanoparticle-receptor affinity was modelled using the attractive branch of a 12-6 Lennard-Jones potential. This attractive potential between the nanoparticles and 'receptor' membrane beads was cutoff at $r_c = 3.75\sigma$. Therefore for each $r < r_c$, the 12-6 Lennard-Jones potential between nanoparticle and receptors is:

$$U_{np/r} = 4\epsilon \left[\left(\frac{r_0}{r}\right)^{12} - \left(\frac{r_0}{r}\right)^{6} \right] \quad (2)$$

where ϵ is the depth of the potential well, r_0 the distance at which the potential is zero, and r the distance between particles.

For the membrane mechanics, we adopted a meshless model. Meshless models can capture membrane topological changes and time dynamics naturally when coupled with MD simulations. However, a careful choice of potential between membrane beads is essential to ensuring a faithful replication of biologically relevant membrane properties. We employed the soft-core pairwise inter-particle potential developed by Yuan, et. al. [54]. A 4-2 Lennard-Jones (LJ) type potential was used for the repulsive branch of the potential

that ensured particle volume exclusion and a cosine function potential was used for the attractive branch of the potential for driving membrane self-assembly. The potential was used for interactions between and among membrane and receptor beads. The molecular dynamics simulations were carried out in the NVE ensemble, where N is the total number of system particles, V is the volume of the simulation box and E is the total energy of the system. A Langevin thermostat was applied to the system components to model interactions with an implicit background solvent. The simulations were typically carried out for 2,000,000 time steps, with a time step of 0.01ŁŁ (where ŁŁ is the MD unit of time). The simulations were implemented with the LAMMPS package [55].

Immuno-fluorescence Polarised bEnd3 were washed twice with PBS, fixed in 4% (w/v) paraformaldehyde (PFA) for 15 minutes, permeabilised with 0.1% (w/v) Triton X-100 in PBS for 10 minutes and incubated with 5% (w/v) BSA in PBS for 1 hour at room temperature. Afterwards, cell monolayers were incubated with primary antibodies diluted in 1% (w/v) BSA and 0.01% (w/v) Triton X-100 in PBS overnight at 4 °C, followed by washing with PBS and incubation with the corresponding secondary antibodies for 2 hours at room temperature. Nuclei was counterstained by incubation with DAPI for 10 minutes. Transwell membranes were excised using a scalpel and mounted on coverslips with Vectashield Mounting Media. Antibodies used in our studies are listed on **Table S1**.

Competition assay For the compeptition assay, FITC-angiopep-2 was dissolved in ultrapure water at 1.75 pM, which is the concentration equivalent to the ligand functionalisation inA_{22}-P. Angiopep-2 or Cy5-labelled A_{22}-P were added separately or together to the apical side of the transwell and cells were incubated at 37 °C. After 10 and 60 minutes, cells were washed twice with PBS and fixed in 4% PFA. Transwell membranes were excised with a scapel and mounted on coverslips using VectaShield Mounting Medium with DAPI for confocal imaging. Quantification of intracellular fluorescence of Cy5-labelled A_{22}-P or FITC was performed *via* ImageJ normalising fluorescence to the number of cell nuclei (DAPI) (**Fig.S6**).

Small molecule pharmacological inhibitors Polarised bEnd3 cells were incubated with CellMask^{TM} Deep Red for 10 minutes at 37 °C and then rinsed with PBS. Dynasore (40 μM) was added to the cell media and pre-incubated for 10 minutes at 37 °C, followed by the addition of A_{22}-P (100 μg mL^{-1}) for 60 minutes. After incubation, cells were washed and media was replaced with serum free FluoroBrite DMEM. For the dynasore recovery experiments, cells were washed with PBS at pH5 before the addition of the imaging media. In the inhibition experiments with N-ethylmaleimide (NEM), cells were incubated with NEM (0.5 mM) for 5 minutes before the addition of the A_{22}-P (100 μg mL^{-1}) for 60 minutes. Live-cell imaging was performed using Leica TCS SP8 confocal microscope (**Fig.S6**).

Membrane cholesterol depletion Prior to the cholesterol depletion assay, media of polarised bEnd3 was changed to serum-free DMEM. Methyl-β-cyclodextrin (CD, 10 mM) was added either to the apical or basal side of the transwell and cells were incubated for 15 minutes at 37 °C. Apical and basal media was collected and used for the quantification of free cholesterol. A_{22}-P (100 μg mL^{-1}) were added to the apical side of the transwell and incubated for 60 minutes at 37 °C. Afterwards, cells were washed twice with PBS and fixed in 4% PFA for 15 minutes, followed by incubation with 0.3% (w/v) Triton X-100 and 10% BSA in PBS for 30 minutes. Cells were incubated with anti-caveolin-1 antibody for 2 hours at room temperature followed by incubation with an appropriate secundary antibody. Cholesterol quantification in the media was performed by using a colorimetric cholesterol quantification kit according to the supplier's instructions (**Fig.S7**).

Lentiviral shRNA silencing Short hairpin RNA (shRNA) lentiviral particles for syndapin-2 were used according to supplier's instructions. Briefly, bEnd3 cells were seeded on a 6-well plate at a density of 100,000 cells per well and grown overnight. At 50% of confluence, cells were treated with the shRNA lentiviral particles in DMEM supplemented with polybrene (5 μg mL^{-1}) and then, incubated overnight. On the next day, the media was replaced, and cells were further incubated for 2 days. Stable clones expressing shRNA were selected by puromycin (5 μg mL^{-1}) and silencing of syndapin-2 was then confirmed by Western blot. Control shRNA lentiviral particles were used as a negative control (**Fig.S8**).

Transmission electron microscopy of brain tissue Brains from mice treated with PtA2-loaded POs were cut into 1x1x1 mm size cubes. Brain tissue was fixed in fresh 3% (v/v) glutaradehyde in phosphate buffer (0.1 M) overnight at 4 °C, and then washed twice in phosphate buffer (0.1 M) at 4 °C at 30 minutes intervals. Samples were dehydrated through a series of ethanol incubations: 75% (15 minutes), 95% (15 minutes), 100% (15 minutes) and 100% (15 minutes) and then placed in an intermediate solvent (propylene oxide) for 2 changes of 15 minutes. Resin infiltration was obtained by placing the specimens in a 50:50 mixture of

propylene/araldite resin leaving the samples with the mixture overnight at room temperature. Afterwards, speciments were moved into Araldite resin for 6-8 hours at room temperature with a change of resin after 3-4 hours. Finally, specimens were embedded in fresh Araldite resin for 48-72 hours at 60 °C. Semi-thin sections of approximately 0.5 μm were cut on a Leica Ultramicrotome and stained with 1% Toluidine blue in Borax. Ultra-thin sections of 70-90 nm tick were cut on a Leica Ultramicrotome and stained for 25 minutes with saturated aqueous uranyl acetate followed by staining with Reynold's lead citrate for 5 minutes. Sections were examined using FEI Tecnai TEM at an accelerating voltage of 80kVv. Electron micrographs were taken using a Gatan digital camera (**Fig.S9**).

Table S1 List of antibodies.

Antibody	Dilution	Supplier, Catalogue Number
Goat polyclonal to PECAM-1	1:200	RD Systems, AF3628
Rabbit polyclonal to claudin-5	1:100	Abcam, ab15106
Rabbit polyclonal to ZO-1	1:100	Abcam, ab221547
Mouse monoclonal to LRP1	1:1000	Abcam, ab28320
Rabbit monoclonal to LRP1 †	1:1000	Abcam, ab92544
Mouse monoclonal to GAPDH †	1:1000	Abcam, ab8245
Rabbit polyclonal to syndapin-2 †	1:400	Abcam, ab37615
Rabbit polyclonal to caveolin-1	1:100	Sigma-Aldrich, C4490
Rabbit polyclonal to clathrin	1:1000	Abcam, ab21679
Rabbit polyclonal to dynamin-2	1:300	Abcam, ab3457
Rabbit polyclonal to EEA-1	1:100	Abcam, ab2900
Rabbit polyclonal to Rab5	1:100	Abcam, ab13253
Rabbit monoclonal to Rab7	1:100	Abcam, ab137029
Rabbit polyclonal to Rab11	1:100	Abcam, ab3612
Rabbit polyclonal to LAMP-1	1:100	Abcam, ab24170
Rabbit polyclonal to β-actin	1:100	Abcam, ab8227
Rabbit monoclonal to myosin	1:200	Abcam, ab92721
Rabbit polyclonal to α-tubulin	1:100	Abcam, ab18251
Rabbit polyclonal to dynein	1:200	Abcam, ab236594
Rabbit polyclonal to kinesin	1:500	Abcam, ab62104
Alexa Fluor 488 goat anti-mouse IgG	1:500	Biolegend, 405319
Alexa Fluor 647 donkey anti-rabbit IgG	1:500	Biolegend, 406414
Alexa Fluor 488 donkey anti-rabbit IgG	1:500	Biolegend, 406416
Alexa Fluor 488 donkey anti-goat Ig	1:500	Abcam, ab150129
Dylight 800 goat anti-mouse IgG †	1:5000	Thermo Fisher Scientific, SA535521
Dylight 800 goat anti-rabbit IgG †	1:5000	Thermo Fisher Scientific, SA535571

† Antibodies used for western blot.

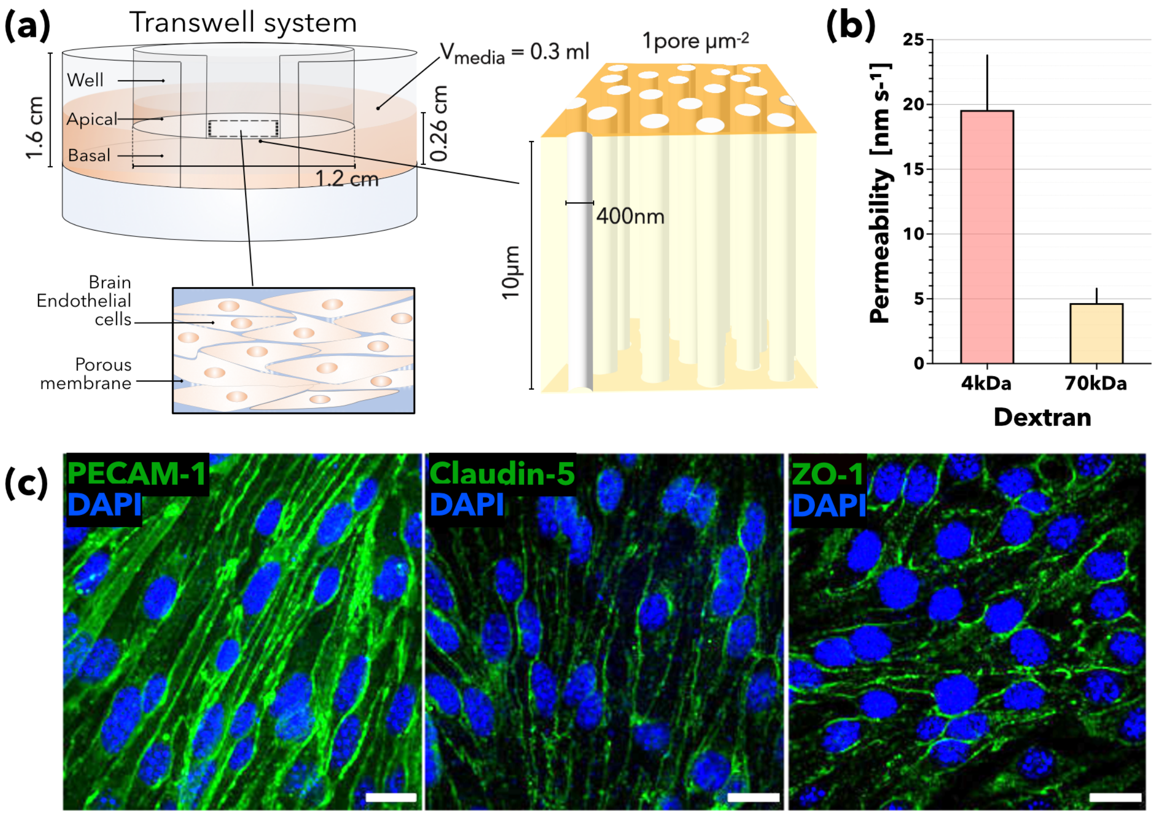

Figure S1: **Characterisation of the *in vitro* BBB model.** Schematic of the *in vitro* model of BBB used to assess transcytosis **(a)**. Bar chart showing the apparent permeability coefficient (P) of 4 and 70 kDa dextrans across bEnd3. Data represented as mean ± SD (n = 9) **(b)**. Confocal images of polarised bEnd3 showing expression of PECAM-1, claudin-5 and ZO-1. PECAM-1, claudin-5 and ZO-1 are shown in green and cell nuclei stained with DAPI (blue). Scale: 20 nm **(c)**.

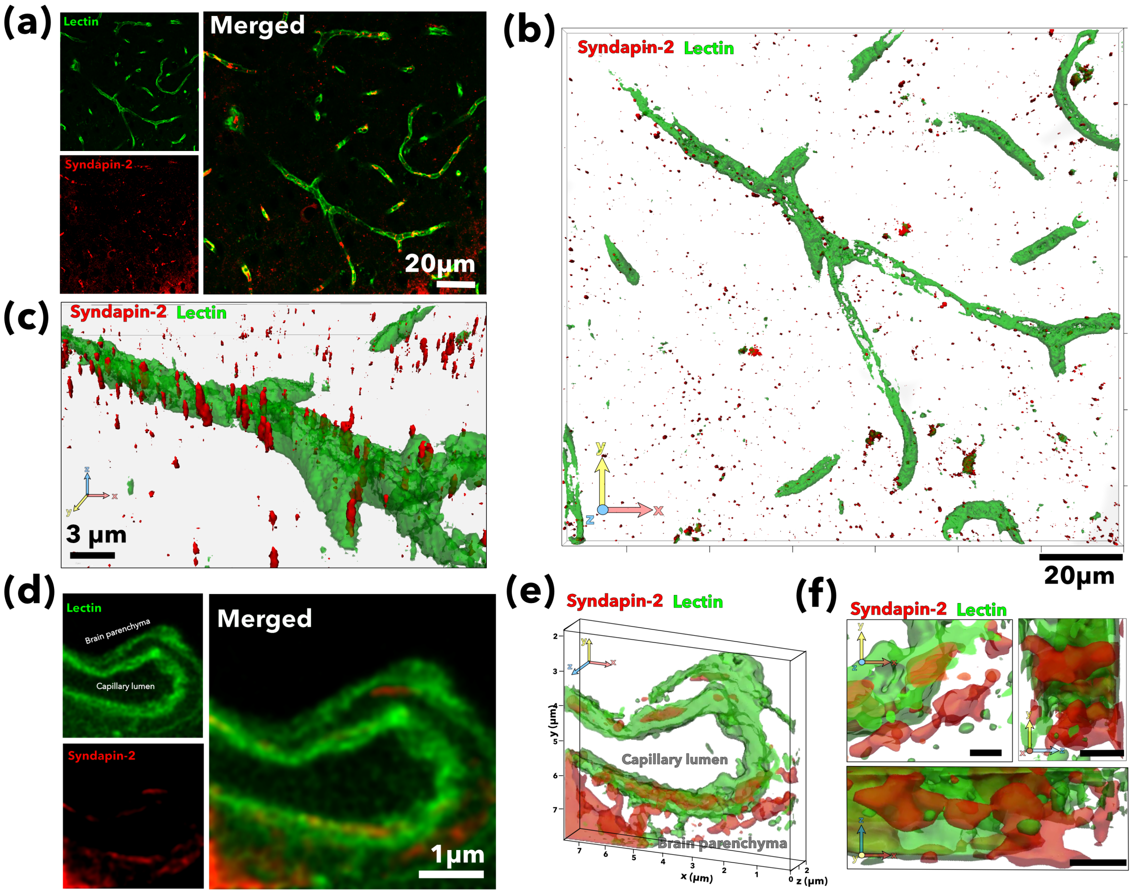

Figure S2: **Syndapin-2 expression *in vivo***. Mouse brain cortex histologies imaged by confocal laser scanning microscopy with capillaries stained by lectin (green) and showing expression of syndapin-2 (red) **(a)**. 3D renderings of brain capillary stained by lectin (green) and syndapin-2 (anti-IgG in red) shown as top view **(b)** and close-up projections **(c)**. Section of a single brain capillary stained by lectin (green) and syndapin-2 (anti-IgG in red) imaged by Stimulated Emission Depletion (STED) microscopy with x-y spatial resolution of c.a. 50 nm **(d)** and corresponding 3D rendering showed as projection **(e)** and close-up from top, bottom and side view **(f)**.

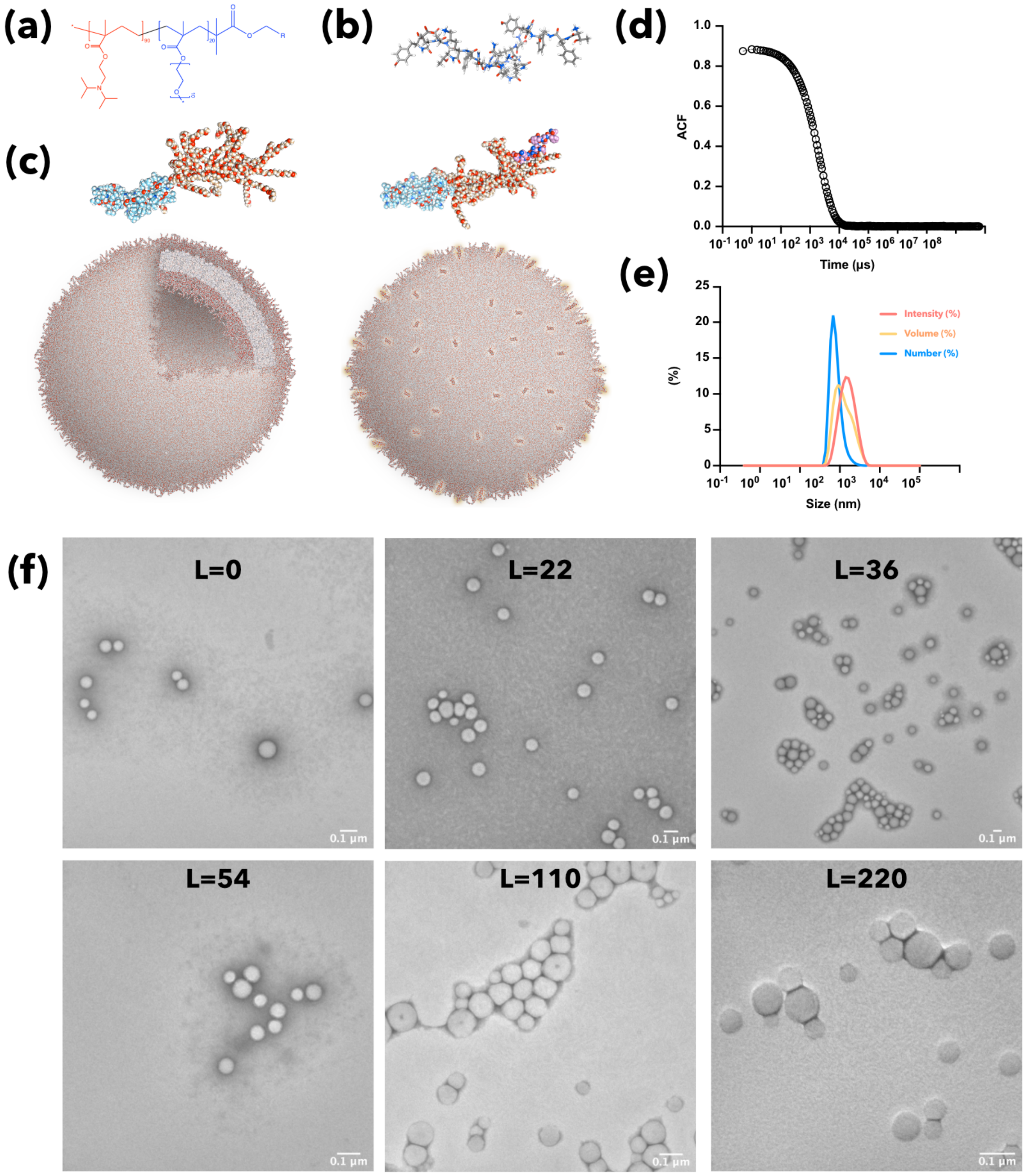

Figure S3: **Characterisation of A$_L$-POs.** Schematic representation of POEGMA$_{20}$-PDPA$_{100}$ **(a)**, angiopep-2 **(b)**, and self-assembled pristine (left) and A$_L$-POs (right) **(c)**. Representative correlogram **(d)** and histogram showing size distribution expressed as percentage of intensity (red), volume (yellow) and number (blue) **(e)** of the A$_L$-POs. Data obtained by dynamic light scattering. Transmission electron micrographs of A$_L$-P with L = 0, 22, 36, 54, 110 and 220 **(f)**.

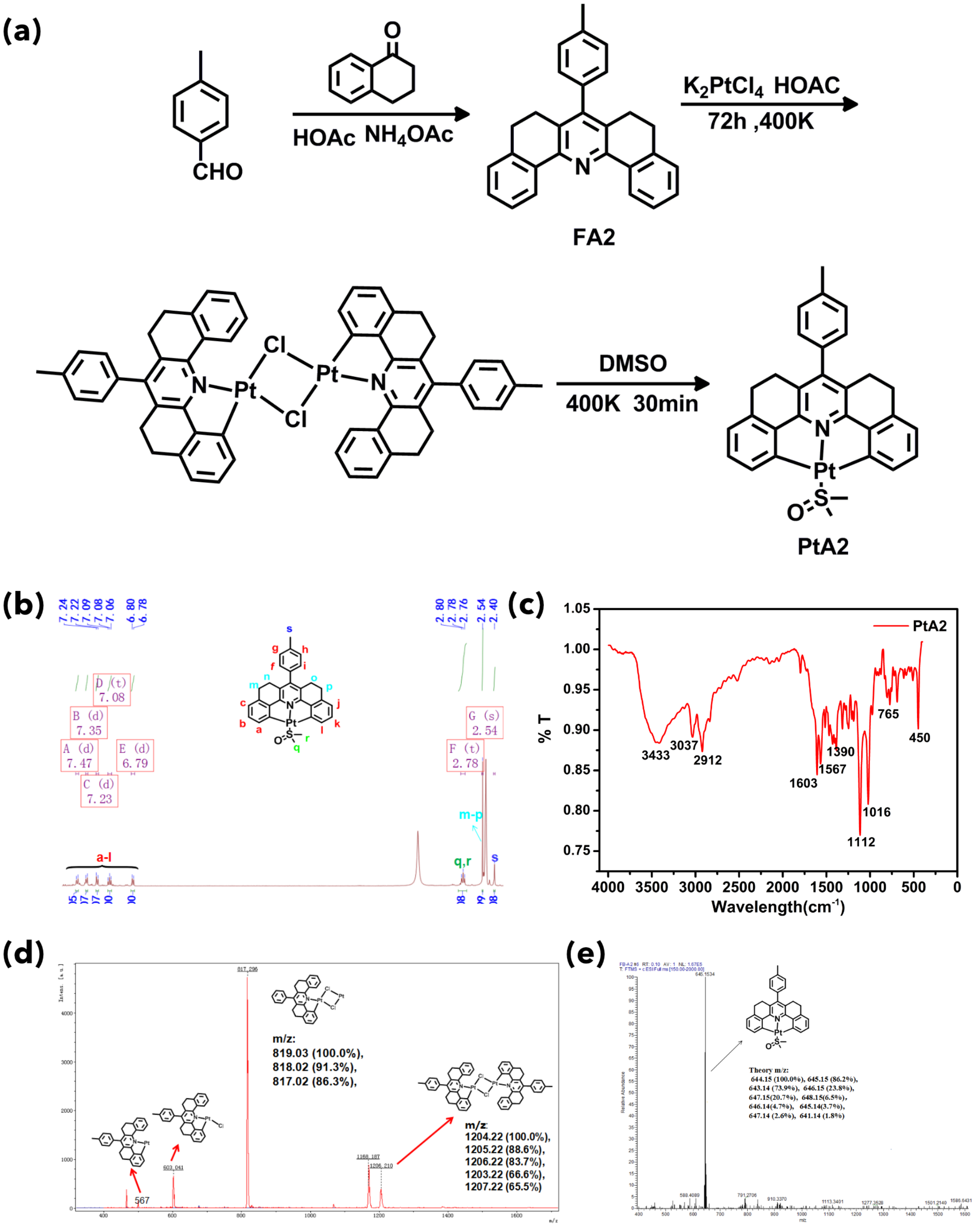

Figure S4: **Characterisation of PtA2.** A schematic representation of the synthesis of PtA2 **(a)**. ^{1}H-NMR **(b)**, FTIR **(c)** and ESI-MS **(d,e)** spectra of purified PtA2.

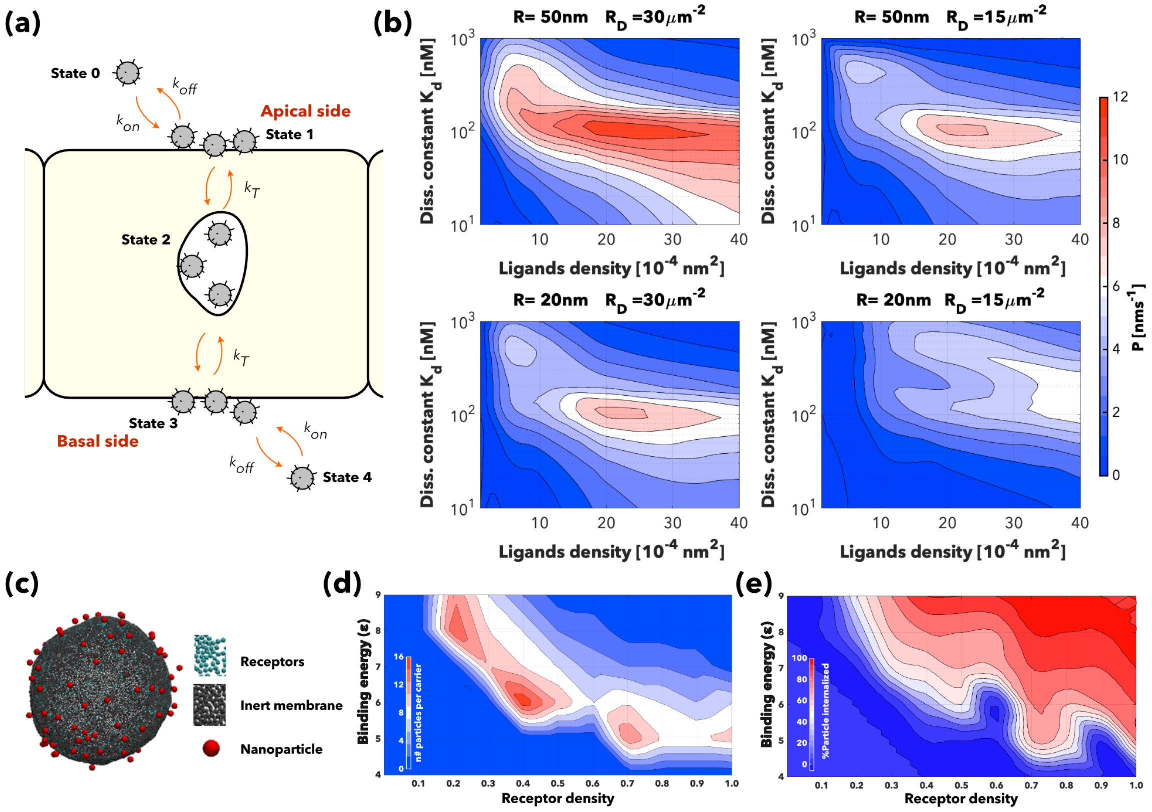

Figure S5: **Modelling of the transcytosis of A$_L$-POs.** Representation of a theoretical model of transcytosis across endothelial cells showing five major steps: binding, endocytosis, trafficking, exocytosis and unbinding **(a)**. Apparent permeability (P) of POs plotted as function of both ligand number per particle (L) and the single ligand/receptor dissociation constant (K_d) for nanoparticles with a radius R of 20 and 50 nm as well as receptor density R_D of 15 and 30 μm^{-2} **(b)**. Simulations of the effect of avidity on membrane topological changes and nanoparticle aggregation dynamics. Coarse-grained membrane model used in the molecular dynamics simulations. Different beads were used to represent a membrane surface patch with no receptors ('inert bead' in black) and membrane surface patch with receptors ('receptor bead' in cyan). Nanoparticles are represented in red **(c)**. Number of particles per carrier **(d)** and percentage of particle internalised **(e)** plotted as function of binding energy and receptor density.

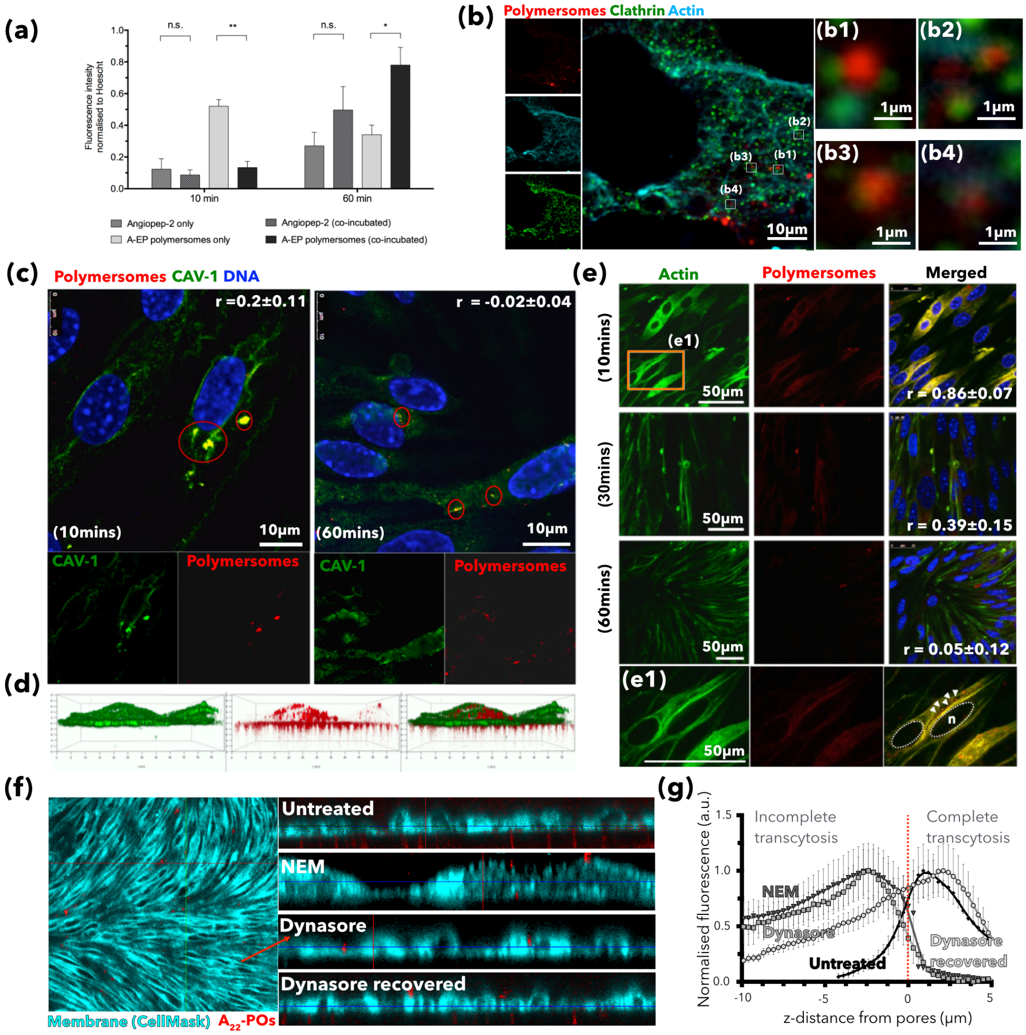

Figure S6: **Transcytosis mechanism of A_{22}-P across brain endothelial cells.** Competition assay of angiopep-2 and A_{22}-P. Comparison of intracellular fluorescence intensity of angiopep-2 or A_{22}-P when incubated alone or co-incubated with polarised bEnd3. Data represented as mean ± SD (n = 3). * P<0.05 and ** P<0.01 **(a)**. Confocal image of clathrin (in green), Cy5-labelled A_{22}-P (in red) and actin (in cyan) in bEnd3 treated with Cy5-labelled A_{22}-P **(b)**. Higher magnification of clathrin (in green) and Cy5-labelled A_{22}-P (in red) staining on bEnd3 showing a partial colocalisation **(b1-b4)**. Confocal images of polarised bEnd3 incubated with Cy5-labelled A_{22}-P (in red) for 10 and 60 minutes and stained for caveolin-1 (in green). Cell nuclei is stained with DAPI (blue). Colocalisation values r are displayed on the top corner of each image **(c)**. 3D rendering of bEnd3 incubated with Cy5-labelled A_{22}-P (in red) and stained for caveolin-1 (green) **(d)**. Confocal micrographs of polarised bEnd3 stained for F-actin (Phalloidin in green) and incubated with Cy5-labelled A_{22}-P (in red) for 10, 30 and 60 minutes. Nuclei stained with DAPI (blue). Colocalisation values r for F-actin and Cy5-labelled A_{22}-P shown at the bottom of each merged image **(e)**. A higher magnification image showing colocalisation of Cy5-labelled A_{22}-P and F-actin **(e1)**. 3D rendering of polarised endothelial cells pre-treated with dynasore or N-ethylmaleimide (NEM) and further incubated for 60 minutes with Cy5-labelled A_{22}-P (in red). Cell membrane is represented in cyan by staining with CellMask^{TM} **(f)**. Quantification of Cy5-labelled A_{22}-P fluorescence intensity across confocal z-stack images of endothelial cells before and after pre-treatment with dynasore for 10 minutes. Dynasore recovery condition represents the cells that were washed with PBS after the incubation with dynasore. In the graph, zero represents the beginning of the pores, negative values above the transwell membrane and positive values within the transwell membrane. Data is represented as mean ± SD (n = 3) **(g)**.

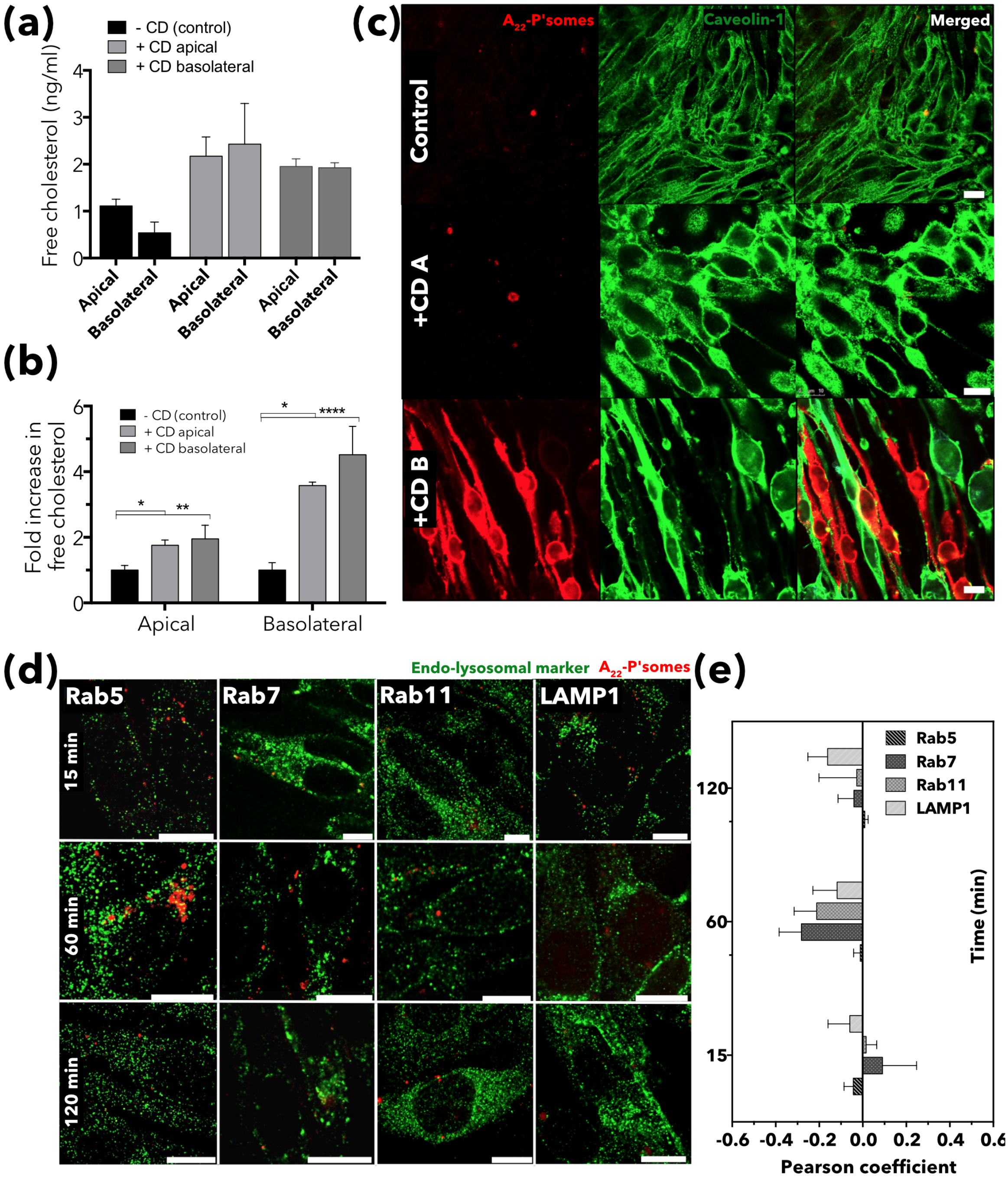

Figure S7: **Cholesterol-dependent and endo-lysosomal independent transcytosis mechanism of A_{22}-POs across brain endothelial cells.** Quantification of free cholesterol released into the media after depletion from the plasma membrane by incubation with methyl-β-cyclodextrin (CD) in the apical or basal side of the transwell. Data is represented as mean ± SD (n = 3) **(a)**. Fold-change in the level of cholesterol in the apical and basal side of the transwell membrane after incubation with CD. Data is represented as mean ± SD (n = 3). * P<0.05, ** P<0.01, **** P<0.0001 **(b)**. Confocal images of Cy5-labelled A_{22}-P (red) in cholesterol-depleted bEnd3 cells either treated with CD in the apical (+CD A) or basal (+CD BL) side of transwell. Caveolin-1 is represented in green. Scale bar: 5μm **(c)**. Confocal images of Cy5-labelled A_{22}-P (red) and markers of endosomal (Rab5, Rab7 and Rab11) and lysosomal (LAMP-1) (in green) endocytic pathways after incubation for 15, 60 and 120 minutes. Scale bar: 10 μm **(d)**. Quantification of the colocalisation of Cy5-labelled A_{22}-P with endosomal and lysosomal markers along the time **(e)**.

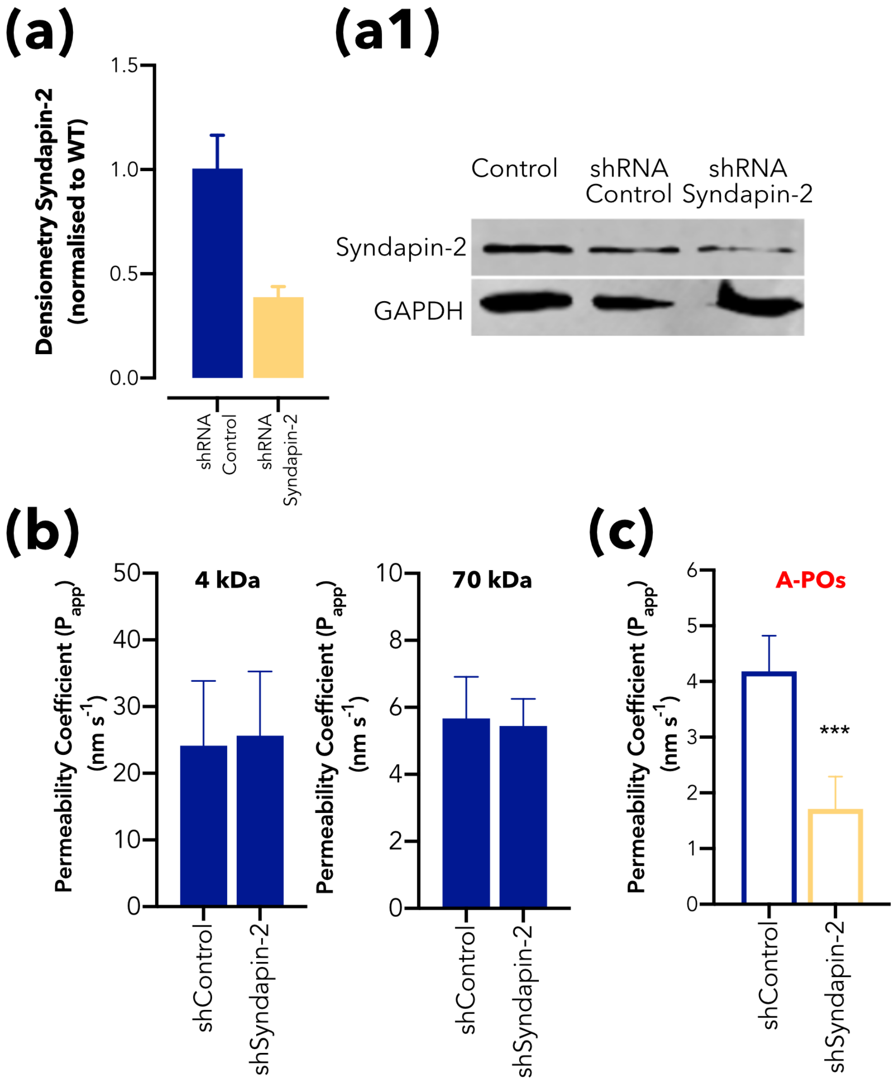

Figure S8: **Syndapin-2 knockdown disrupts transcytosis of A_{22}-POs.** Expression of syndapin-2 on shRNA-transfected bEnd3 cells quantified by western blot. Levels of syndapin-2 on bEnd3 either treated with shRNA control or syndapin-2 were normalised to wild-type cells. Data is represented as mean ± SD (n = 6) **(a)**. Representative blot showing the expression of syndapin-2 and GAPDH (loading control) **(a1)**. Bar charts showing apparent permeability of 4 and 70 kDa dextrans across bEnd3 transfected with shRNA control or shRNA for syndapin-2 knockdown. Data represented as mean ± SD (n = 6) **(b)**. Apparent permeability of Cy7-labelled A_{22}-P across control and syndapin-2 knockout bEnd3 cells. Data represented as mean ± SD (n = 6). *** P<0.001 **(c)**.

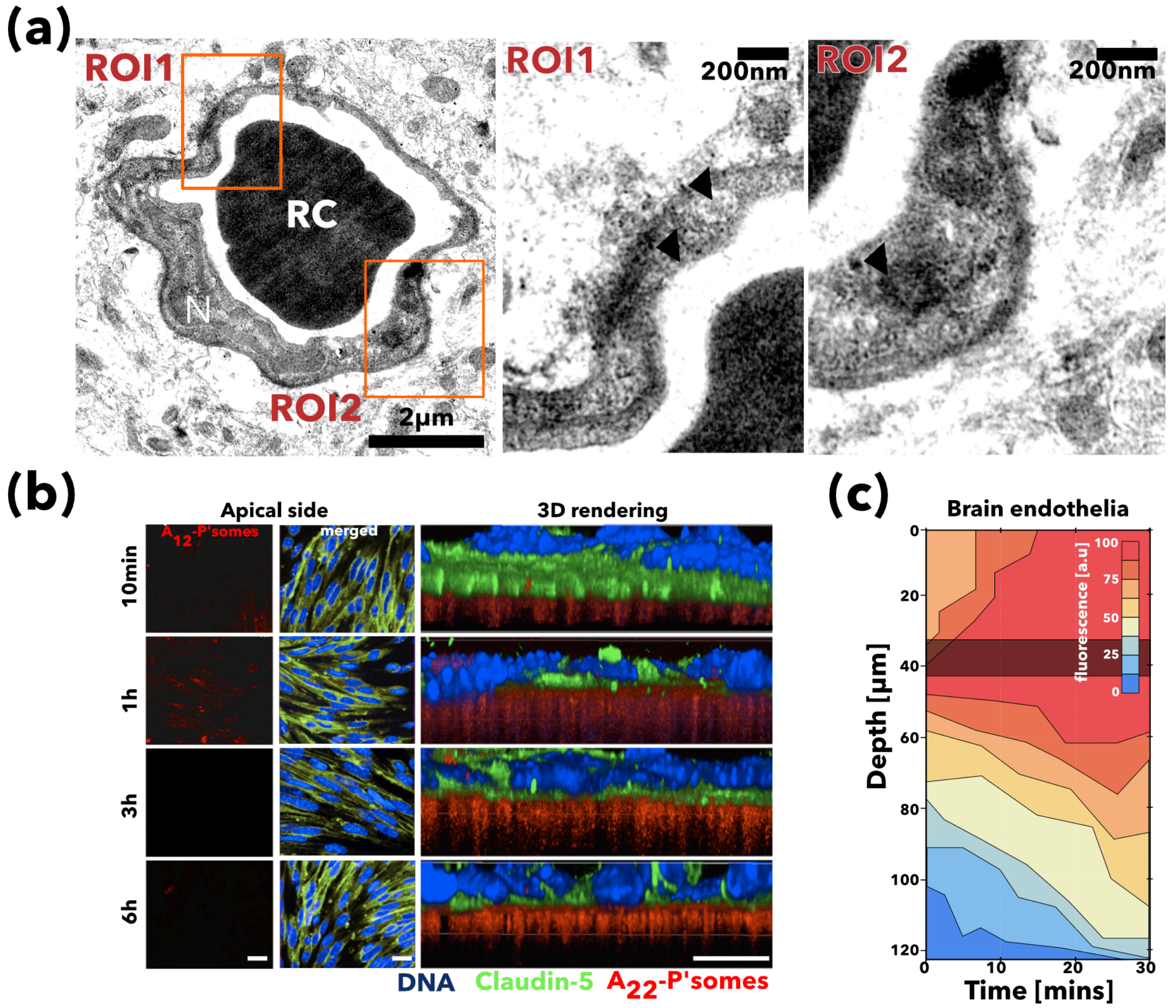

Figure S9: **Penetration of A_{22}-POs across *in vivo* and *in vitro* BBB.** Transmission electron micrograph of PtA2-loaded A_{22}-P penetrating brain capillaries. Two high magnification images show in detail two regions of interest (ROI). RC: red blood cell and N: nucleus **(a)**. Confocal images and corresponding 3D rendering of polarised bEnd3 incubated with Cy5-labelled A_{22}-P (red) for 10 minutes, 1, 3 and 6 hours. Tight junction, claudin-5, is represented in green and the nuclei counterstained with DAPI (in blue). Scale bar: 25 μm **(b)**. Real-time 4D confocal imaging of transcytosis of Cy5-labelled A_{22}-P. Heat map of Cy5-labelled A_{22}-P fluorescence in the extended transwell area over time obtained through 4D imaging **(c)**.

Movie S1: 3D rendering as function of time reconstructed from confocal laser scanning micrographs over 40 minutes of brain endothelial cells exposed to LRP1 targeting A_{22}-POs (red). The cell nucleus is stained with DAPI (blue) and the cell membrane with CellMask (cyan).

Movie S2: Fast (50fps) 3D rendering as function of time reconstructed from confocal laser scanning micrographs collected over 5 minutes of brain endothelial cells exposed to LRP1 targeting A_{22}-POs (red). The cell membrane is stained with CellMask (green).

Movie S3: Top view of a 3D rendering as function of time reconstructed from fast Stimulated Emission Depletion (STED) microscopy micrographs collected over 6 minutes of a section of a single brain endothelial cell exposed to LRP1 targeting A_{22}-POs. The structure emerging from the interaction between the POs are coloured according to their depth expressed in micrometers.

Movie S4: Side view of a 3D rendering as function of time reconstructed from fast Stimulated Emission Depletion (STED) microscopy micrographs collected over 6 minutes of a section of a single brain endothelial cell exposed to LRP1 targeting A_{22}-POs. The structure emerging from the interaction between the POs are coloured according to their depth expressed in micrometers.

Movie S5: Bottom view of a 3D rendering as function of time reconstructed from fast Stimulated Emission Depletion (STED) microscopy micrographs collected over 6 minutes of a section of a single brain endothelial cell exposed to LRP1 targeting A22-POs polymersomes. The structure emerging from the interaction between the POs are coloured according to their depth expressed in micrometers.

REFERENCES AND NOTES

1. G. A. Dienel, Brain glucose metabolism: Integration of energetics with function. *Physiol. Rev.* **99**, 949–1045 (2019).

2. J. Abbott, L. Rönnbäck, E. Hansson, Astrocyte–endothelial interactions at the blood–brain barrier. *Nat. Rev. Neurosci.* **7**, 41–53 (2006).

3. G. Fullstone, S. Nyberg, X. Tian, G. Battaglia, Chapter two-from the blood to the central nervous system: A nanoparticle's journey through the blood–brain barrier by transcytosis. *Int. Rev. Neurobiol.* **130**, 41–72 (2016).

4. Z. Zhao, A. R. Nelson, C. Betsholtz, B. V. Zlokovic, Establishment and dysfunction of the blood-brain barrier. *Cell* **163**, 1064–1078 (2015).

5. W. A. Banks, From blood-brain barrier to blood-brain interface: New opportunities for CNS drug delivery. *Nat. Rev. Drug Discov.* **15**, 275–292 (2016).

6. S. Yazdani, J. R. Jaldin-Fincati, R. V. S. Pereira, A. Klip, Endothelial cell barriers: Transport of molecules between blood and tissues. *Traffic* **20**, 390–403 (2019).

7. Z. M. Qian, H. Li, H. Sun, K. Ho, Targeted drug delivery via the transferrin receptor-mediated endocytosis pathway. *Pharmacol. Rev.* **54**, 561–587 (2002).

8. K. Y. Y. Fung, G. D. Fairn, W. L. Lee, Transcellular vesicular transport in epithelial and endothelial cells: Challenges and opportunities. *Traffic* **19**, 5–18 (2018).

9. H. Emonard, L. Théret, A. H. Bennasroune, S. Dedieu, Regulation of LRP-1 expression: Make the point. *Pathol. Biol.* **62**, 84–90 (2014).

10. A. P. Lillis, L. B. Van Duyn, J. E. Murphy-Ullrich, D. K. Strickland, LDL receptor-related protein 1: Unique tissue-specific functions revealed by selective gene knockout studies. *Physiol. Rev.* **88**, 887–918 (2008).

11. Y. Zhao, D. Li, J. Zhao, J. Song, Y. Zhao, The role of the low-density lipoprotein receptor-related protein 1 (LRP-1) in regulating blood-brain barrier integrity. *Rev. Neurosci.* **27**, 623–634 (2016).

12. P. L. Tuma, A. L. Hubbard, Transcytosis: Crossing cellular barriers. *Physiol. Rev.* **83**, 871–932 (2003).

13. G. Bu, E. A. Maksymovitch, J. M. Nerbonne, A. L. Schwartz, Expression and function of the low density lipoprotein receptor-related protein (LRP) in mammalian central neurons. *J. Biol. Chem.* **269**, 18521–18528 (1994).

14. A. Gaultier, X. Wu, N. Le Moan, S. Takimoto, G. Mukandala, K. Akassoglou, W. M. Campana, S. L. Gonias, Low-density lipoprotein receptor-related protein 1 is an essential receptor for myelin phagocytosis. *J. Cell Sci.* **122**, 1155–1162 (2009).

15. Y. Li, M. P. Marzolo, P. van Kerkhof, G. J. Strous, G. Bu, The Y*XX*L motif, but not the two NP*X*Y motifs, serves as the dominant endocytosis signal for low density lipoprotein receptor-related protein. *J. Biol. Chem.* **275**, 17187–17194 (2000).

16. C. Fillebeen, L. Descamps, M.-P. Dehouck, L. Fenart, M. Benaïssa, G. Spik, R. Cecchelli, A. Pierce, Receptor-mediated transcytosis of lactoferrin through the blood-brain barrier. *J. Biol. Chem.* **274**, 7011–7017 (1999).

17. W. Pan, A. J. Kastin, T. C. Zankel, P. van Kerkhof, T. Terasaki, G. Bu, Efficient transfer of receptor-associated protein (RAP) across the blood-brain barrier. *J. Cell Sci.* **117**, 5071–5078 (2004).

18. M. Demeule, A. Régina, C. Ché, J. Poirier, T. Nguyen, R. Gabathuler, J.-P. Castaigne, R. Béliveau, Identification and design of peptides as a new drug delivery system for the brain. *J. Pharmacol. Exp. Ther.* **324**, 1064–1072 (2008).

19. S. Ayloo, C. Gu, Transcytosis at the blood-brain barrier. *Curr. Opin. Neurobiol.* **57**, 32–38 (2019).

20. Y. J. Yu, Y. Zhang, M. Kenrick, K. Hoyte, W. Luk, Y. Lu, J. Atwal, J. M. Elliott, S. Prabhu, R. J. Watts, M. S. Dennis, Boosting brain uptake of a therapeutic antibody by reducing its affinity for a transcytosis target. *Sci. Transl. Med.* **3**, 84ra44 (2011).

21. J. Niewoehner, B. Bohrmann, L. Collin, E. Urich, H. Sade, P. Maier, P. Rueger, J. O. Stracke, W. Lau, A. C. Tissot, H. Loetscher, A. Ghosh, P.-O. Freskgård, Increased brain penetration and potency of a therapeutic antibody using a monovalent molecular shuttle. *Neuron* **81**, 49–60 (2014).

22. R. Villaseñor, M. Schilling, J. Sundaresan, Y. Lutz, L. Collin, Sorting tubules regulate blood-brain barrier transcytosis. *Cell Rep.* **21**, 3256–3270 (2017).

23. Y. Anraku, H. Kuwahara, Y. Fukusato, A. Mizoguchi, T. Ishii, K. Nitta, Y. Matsumoto, K. Toh, K. Miyata, S. Uchida, K. Nishina, K. Osada, K. Itaka, N. Nishiyama, H. Mizusawa, T. Yamasoba, T. Yokota, K. Kataoka, Glycaemic control boosts glucosylated nanocarrier crossing the BBB into the brain. *Nat. Commun.* **8**, 1001 (2017).

24. M. Simionescu, N. Simionescu, *Endothelial Cell Biology in Health and Disease* (Springer Science & Business Media, 2013).

25. M. Bundgaard, Tubular invaginations in cerebral endothelium and their relation to smooth-surfaced cisternae in hagfish (*Myxine glutinosa*). *Cell Tissue Res.* **249**, 359–365 (1987).

26. D. Feng, J. A. Nagy, J. Hipp, H. F. Dvorak, A. M. Dvorak, Vesiculo-vacuolar organelles and the regulation of venule permeability to macromolecules by vascular permeability factor, histamine, and serotonin. *J. Exp. Med.* **183**, 1981–1986 (1996).

27. B. J. Peter, H. M. Kent, I. G. Mills, Y. Vallis, P. J. G. Butler, P. R. Evans, H. T. McMahon, Bar domains as sensors of membrane curvature: The amphiphysin BAR structure. *Science* **303**, 495–499 (2004).

28. Q. Wang, M. V. A. S. Navarro, G. Peng, E. Molinelli, S. L. Goh, B. L. Judson, K. R. Rajashankar, H. Sondermann, Molecular mechanism of membrane constriction and tubulation mediated by the f-bar protein pacsin/syndapin. *Proc. Natl. Acad. Sci. U.S.A.* **106**, 12700–12705 (2009).

29. A. Frost, R. Perera, A. Roux, K. Spasov, O. Destaing, E. H. Egelman, P. De Camilli, V. M. Unger, Structural basis of membrane invagination by F-BAR domains. *Cell* **132**, 807–817 (2008).

30. X. Tian, S. Nyberg, P. S. Sharp, J. Madsen, N. Daneshpour, S. P. Armes, J. Berwick, M. Azzouz, P. Shaw, N. J. Abbott, G. Battaglia, LRP-1-mediated intracellular antibody delivery to the central nervous system. *Sci. Rep.* **5**, 11990 (2015).

31. X. Tian, O. Brookes, G. Battaglia, Pericytes from mesenchymal stem cells as a model for the blood-brain barrier. *Sci. Rep.* **7**, 39676 (2017).

32. M. Uhlén, L. Fagerberg, B. M. Hallström, C. Lindskog, P. Oksvold, A. Mardinoglu, Å. Sivertsson, C. Kampf, E. Sjöstedt, A. Asplund, I. M. Olsson, K. Edlund, E. Lundberg, S. Navani, C. A.-K. Szigyarto, J. Odeberg, D. Djureinovic, J. O. Takanen, S. Hober, T. Alm, P.-H. Edqvist, H. Berling, H. Tegel, J.

Mulder, J. Rockberg, P. Nilsson, J. M. Schwenk, M. Hamsten, K. von Feilitzen, M. Forsberg, L. Persson, F. Johansson, M. Zwahlen, G. von Heijne, J. Nielsen, F. Pontén, Tissue-based map of the human proteome. *Science* **347**, 1260419 (2015).

33. A. Régina, M. Demeule, C. Ché, I. Lavallée, J. Poirier, R. Gabathuler, R. Béliveau, J.-P. Castaigne, Antitumour activity of ANG1005, a conjugate between paclitaxel and the new brain delivery vector angiopep-2. *Br. J. Pharmacol.* **155**, 185–197 (2008).

34. M. Demeule, N. Beaudet, A. Régina, É. Besserer-Offroy, A. Murza, P. Tétreault, K. Belleville, C. Ché, A. Larocque, C. Thiot, R. Béliveau, J.-M. Longpré, É. Marsault, R. Leduc, J. E. Lachowicz, S. L. Gonias, J.-P. Castaigne, P. Sarret, Conjugation of a brain-penetrant peptide with neurotensin provides antinociceptive properties. *J. Clin. Invest.* **124**, 1199–1213 (2014).

35. S. An, D. He, E. Wagner, C. Jiang, Peptide-like polymers exerting effective glioma-targeted siRNA delivery and release for therapeutic application. *Small* **11**, 5142–5150 (2015).

36. S. Huang, J. Li, L. Han, S. Liu, H. Ma, R. Huang, C. Jiang, Dual targeting effect of angiopep-2-modified, DNA-loaded nanoparticles for glioma. *Biomaterials* **32**, 6832–6838 (2011).

37. A. K. Apawu, S. M. Curley, A. R. Dixon, M. Hali, M. Sinan, R. D. Braun, J. Castracane, A. T. Cacace, M. Bergkvist, A. G. Holt, Mri compatible MS2 nanoparticles designed to cross the blood-brain-barrier: Providing a path towards tinnitus treatment. *Nanomedicine* **14**, 1999–2008 (2018).

38. A. Joseph, C. Contini, D. Cecchin, S. Nyberg, L. Ruiz-Perez, J. Gaitzsch, G. Fullstone, J. Azizi, J. Preston, G. Volpe, G. Battaglia, Active delivery to the brain by chemotaxis. bioRxiv 061325 [**Preprint**]. 29 June 2016. https://doi.org/10.1101/061325.

39. E.-S. Kim, D. Kim, S. Nyberg, A. Poma, D. Cecchin, S. A. Jain, K.-A Kim, Y.-J. Shin, E.-H. Kim, M. Kim, S.-H. Baek, J.-K. Kim, T. R. Doeppner, A. Ali, J. Redgrave, G. Battaglia, A. Majid, O.-N. Bae, LRP-1 functionalized polymersomes enhance the efficacy of carnosine in experimental stroke. *Sci. Rep.* **10**, 699 (2020).

40. L. Messager, J. Gaitzsch, L. Chierico, G. Battaglia, Novel aspects of encapsulation and delivery using polymersomes. *Curr. Opin. Pharmacol.* **18**, 104–111 (2014).

41. J. D. Robertson, G. Yealland, M. Avila-Olias, L. Chierico, O. Bandmann, S. A. Renshaw, G. Battaglia. pH-sensitive tubular polymersomes: Formation and applications in cellular delivery. *ACS Nano* **8**, 4650–4661 (2014).

42. L. Ruiz-Pérez, L. Messager, J. Gaitzsch, A. Joseph, L. Sutto, F. L. Gervasio, G. Battaglia, Molecular engineering of polymersome surface topology. *Sci. Adv.* **2**, e1500948 (2016).

43. C. A. Puckett, R. J. Ernst, J. K. Barton, Exploring the cellular accumulation of metal complexes. *Dalton Trans.* **39**, 1159–1170 (2010).

44. E. Baggaley, J. A. Weinstein, J. A. G. Williams, Lighting the way to see inside the live cell with luminescent transition metal complexes. *Coord. Chem. Rev.* **256**, 1762–1785 (2012).

45. C. Greenough, S. Coakley, M. Holcombe, M. Kiran, L. S. Chin, D. J. Worth, "Flame: An approach to the parallelisation of agent-based applications" (Science and Technology Facilities Council Technical Report RAL-TR-2012-013, RAL Library STFC Rutherford Appleton Laboratory, 2012).

46. X. Tian, S. Angioletti-Uberti, G. Battaglia, On the design of precision nanomedicines. *Sci. Adv.* **6**, eaat0919 (2020).

47. Y. Bertrand, J.-C. Currie, M. Demeule, A. Régina, C. Ché, A. Abulrob, D. Fatehi, H. Sartelet, R. Gabathuler, J.-P. Castaigne, D. Stanimirovic, R. Béliveau, Transport characteristics of a novel peptide platform for CNS therapeutics. *J. Cell. Mol. Med.* **14**, 2827–2839 (2010).

48. A. Šarić, A. Cacciuto, Mechanism of membrane tube formation induced by adhesive nanocomponents. *Phys. Rev. Lett.* **109**, 188101 (2012).

49. F. Xia, X. Gao, E. Kwan, P. P. L. Lam, L. Chan, K. Sy, L. Sheu, M. B. Wheeler, H. Y. Gaisano, R. G. Tsushima, Disruption of pancreatic β-cell lipid rafts modifies $k_v2.1$ channel gating and insulin exocytosis. *J. Biol. Chem.* **279**, 24685–24691 (2004).

50. P. Virtanen, R. Gommers, T. E. Oliphant, M. Haberland, T. Reddy, D. Cournapeau, E. Burovski, P. Peterson, W. Weckesser, J. Bright, S. J. van der Walt, M. Brett, J. Wilson, K. J. Millman, N. Mayorov, A. R. J. Nelson, E. Jones, R. Kern, E. Larson, C. J. Carey, İ. Polat, Y. Feng, E. W. Moore, J. V. Plas, D. Laxalde, J. Perktold, R. Cimrman, I. Henriksen, E. A. Quintero, C. R. Harris, A. M. Archibald, A. H.

Ribeiro, F. Pedregosa, P. van Mulbregt; SciPy 1.0 Contributors, SciPy 1.0: Fundamental algorithms for scientific computing in python. *Nat. Methods* **17**, 261–272 (2020).

51. R. G. Bacabac, T. H. Smit, S. C. Cowin, J. J. W. A. Van Loon, F. T. M. Nieuwstadt, R. Heethaar, J. Klein-Nulend, Dynamic shear stress in parallel-plate flow chambers. *J. Biomech.* **38**, 159–167 (2005).

52. R. N. Alyaudtin. A. Reichel, R. Löbenberg, P. Ramge, J. Kreuter, D. J. Begley, Interaction of poly(butylcyanoacrylate) nanoparticles with the blood-brain barrier in vivo an *in vitro*. *J. Drug Target.* **9**, 209–221 (2001).

53. U. Schröder, B. A. Sabel, Nanoparticles, a drug carrier system to pass the blood-brain barrier, permit central analgesic effects of i.v. dalargin injections. *Brain Res.* **710**, 121–124 (1996).

54. H. Yuan, C. Huang, J. Li, G. Lykotrafitis, S. Zhang, One-particle-thick, solvent-free, coarse-grained model for biological and biomimetic fluid membranes. *Phys. Rev. E* **82**, 011905 (2010).

55. S. Plimpton, Fast parallel algorithms for short-range molecular dynamics. *J. Comput. Phys.* **117**, 1–19 (1995).
